# The decriminalization of illicit drugs in British Columbia: a national evaluation protocol

**DOI:** 10.1186/s12889-024-20336-9

**Published:** 2024-10-18

**Authors:** Cayley Russell, Farihah Ali, Sameer Imtiaz, Amanda Butler, Alissa Greer, Jürgen Rehm, the Decriminalization Evaluation Research Group, Geoff Bardwell, Matthew Bonn, Jade Boyd, Julie Bruneau, Jean Costello, Frank Crichlow, Jean-François Crépault, Louisa Degenhardt, Tara Elton-Marshall, Sarah Ferencz, Tara Gomes, João Castel-Branco Goulão, Paul N Griffiths, Matthew Hickman, David C. Hodgins, Kate Hodgson, Elaine Hyshka, Bernard Le Foll, Rennie Linklater, Kurt Lock, Sean Patenaude, Laura M. Mackinnon, Taija McLuckie, Sanjana Mitra, Michael Nurse, Kali-olt Sedgemore, Rita Shahin, Wayne M. Smith, Sherry H. Stewart, Dan Werb, Jessica C. Xavier

**Affiliations:** 1 Institute for Mental Health Policy Research, Centre for Addiction and Mental Health (CAMH), 250 College St., Toronto, ON M5T 1R8 Canada; 2 Ontario Node, Canadian Research Initiative in Substance Matters (CRISM), 250 College St., Toronto, ON M5T 1R8 Canada; 3 School of Criminology, Simon Fraser University (SFU), 8888 University Drive, Burnaby, BC V5A 1S6 Canada; 4 Department of Psychiatry, University of Toronto, 1, King’s College Circle, Toronto, ON M5S 1A8 Canada; 5 Institute of Medical Science (IMS), University of Toronto, 1, King’s College Circle, Toronto, ON M5S 1A8 Canada; 6 Dalla Lana School of Public Health, University of Toronto, 155 College St., Toronto, ON M5T 3M7 Canada; 7 Campbell Family Mental Health Research Institute, Centre for Addiction and Mental Health (CAMH), 1001 Queen St. West, Toronto, ON M6J 1H4 Canada; 8 Center for Interdisciplinary Addiction Research (ZIS), Department of Psychiatry and Psychotherapy, University Medical Center Hamburg-Eppendorf (UKE), Martinistraße 52, Hamburg, 20246 Germany

**Keywords:** Addiction, Canada, Decriminalization, Drug Policy, Evaluation, Public Health

## Abstract

**Background:**

On January 31st, 2023, the province of British Columbia (BC), Canada, was granted a federal exemption allowing adults (aged 18 +) to possess up to 2.5 g of select illicit drugs. The exemption will be in place for three years (2023–2026), marking the first formal decriminalization of illicit drug policy reform in Canada. BC’s decriminalization initiative is premised on several goals. This project seeks to evaluate each of these goals and their individual and combined contributions to determine the overall success of this policy.

**Methods:**

The following protocol paper provides a detailed outline of a five-year (2022-2027) national evaluation of BC’s decriminalization initiative, as well as the specific objectives, methodologies, and planned analyses for eight interrelated sub-studies that comprise the evaluation. These sub-studies fall under the following five topical areas of research: 1) people who use drugs (PWUD), 2) the police and the criminal justice system, 3) the general public, 4) the health services system, and 5) an economic analysis. Additional research activities may also be explored.

**Results:**

The overall evaluation and specific sub-study designs were informed by intensive stakeholder engagement. The evaluation was developed in collaboration with an international expert committee who came together to undertake a nominal group technique to decide on the final evaluation design and corresponding logic model. The evaluation will also employ an advisory board and individual sub-study working groups comprised of experts and PWUD who will oversee the development and implementation of the overall evaluation as well as each sub-study.

**Discussion:**

This evaluation will draw on implementation science research practices to evaluate and understand the full impacts of this novel drug policy experiment. Results will be widely disseminated through manuscripts, reports, presentations, and infographics, which will be adapted and tailored for specific audiences. The protocol identifies several anticipated challenges and limitations. This evaluation’s evidence-based findings will be poised to offer pivotal insights that can shape and refine the discourse on drug policy and will serve as a critical resource for understanding the multifaceted impacts of decriminalization.

**Supplementary Information:**

The online version contains supplementary material available at 10.1186/s12889-024-20336-9.

## Background

In Canada, drug use is prohibited by law under the *Controlled Drugs and Substances Act*, which is the overarching federal law that regulates the possession, production, distribution, and sale of controlled drugs and classifies them based on their perceived potential for harm and misuse [1]. The *Controlled Drugs and Substances Act* can be amended on a case-by-case basis if it is determined that an exemption is required in the interest of emerging health threats, the public interest, or based on updated medical/scientific evidence [2]. As an example, in 2018, Canada amended the *Controlled Drugs and Substances Act* to remove cannabis and subsequently established the Cannabis Act, essentially legalizing recreational cannabis use and possession. This move was a marked departure from over a century of prohibition, and considered progressive as Canada is the first economically advanced country to legalize cannabis at the federal level [3, 4]. More recently, in 2022, the province of British Columbia (BC) was granted an exemption to the *Controlled Drugs and Substances Act* permitting adults (aged 18 +) to possess up to 2.5 g of certain illicit drugs, including opioids, methamphetamine, cocaine/crack-cocaine, and MDMA (ecstasy) [5, 6]. The impetus for this regulatory change was the ongoing overdose death crisis that has gripped the nation for a number of years, and disproportionately affected the province of BC [7]. The exemption is a three-year pilot program that will be in effect from January 31st 2023 until January 31st, 2026 [8]. This drug policy reform represents the latest monumental shift from the historical prohibition of illegal drugs in Canada, and places Canada amongst other countries that have implemented similar decriminalization initiatives [9]. Notably, the exemption is specific to the province of BC but other Canadian provinces and cities have submitted similar applications or have expressed their intent to [10].

The concept of decriminalizing illicit drugs is not new and various decriminalization initiatives have been adopted internationally over the past few decades. Jurisdictions such as Portugal, Oregon (USA), Paraguay, Bolivia, Mexico, Poland, and Germany have introduced diverse decriminalization strategies [11–16]. However, the implementation, outcomes, and levels of success of decriminalization have varied among these locations [17]. For instance, in 2001, Portugal decriminalized the personal possession of all illegal drugs as part of a wider re-orientation of drug policy towards a public health-focused approach where drug possession began to be treated as an administrative offence instead of a criminal one, resulting in fines or community service in place of arrests or incarceration [18–20]. The Portuguese model gained widespread recognition for its success, initially resulting in higher rates of treatment, lower HIV transmission rates, stable drug prices, continued low overdose rates, and a decrease in incarceration rates [12–15]. Yet, more recently, Portugal has come under scrutiny related to a reversal in some of these trends, including increases in overdose rates, public visibility of drug use, and wait times for treatment, as well as a subsequent decrease in treatment referrals and uptake [21–23]. Some of these trends have also been observed in other European Union countries with varying drug decriminalization policies [24]. These data have been used to cast doubt by some on the effectiveness of decriminalization initiatives [25, 26] These doubts have been further cemented by the situation in Oregon, which, in 2020, was the first state in the United States to decriminalize illicit drugs [26]. Just over two years in, in light of mixed evidence regarding whether decriminalization has been associated with changes in fatal drug overdose rates [27, 28], critics have emphasized that public visibility of drug use is at an all-time high [28] and have panned the initiative as a failure [29, 30]. The policy was subsequently repealed as of September 1 2024 [31]. The extent to which these observations are causally linked to decriminalization is unclear given the possibility of external factors which may have impacted the drug supply, drug consumption patterns, and the visibility of drug use, such as the novel coronavirus disease pandemic (COVID-19) and associated public health measures and disruptions to the drug supply [32–35].

Assessing the effectiveness and ultimate success of decriminalization initiatives depends on the goal(s) of the policy and the underlying model(s) on which they are premised. Among the countries that have implemented decriminalization initiatives, there exists significant diversity in these goals and models. For example, some models have introduced administrative fines as penalties, while others specify drug confiscation, yet others enforce either voluntary or mandatory treatment for people found in possession of drugs. Additionally, some models have established defined threshold amounts for each drug, whereas others draw distinctions between ‘personal possession’ and ‘trafficking’ based on the discretion of law enforcement or court officials [9]. Further, the implementation of adjunct social and health supports and funding varies considerably. Discrepancies across policies and local conditions complicate the comparison of outcomes and judgments about whether decriminalization of drugs is ultimately ‘effective’.

BC’s decriminalization model’s primary goal is to address criminalization as a social determinant of health and reduce harms caused by criminalization by removing structural barriers to critical health and social services to support people who use drugs (PWUD) [8]. Concretely, the goals of the initiative, as stated in the government’s application, are multi-faceted and include the reduction in drug seizures, arrests, charges, penalties, and criminal records for simple possession and associated court time and resources, the reduction of health, social, and economic harms associated with criminalization, the reduction of stigma, and the reduction of illicit drug poisoning deaths [8]. Additionally, the stated goals include an increase in access to and engagement in health and social services, improved interactions and trust between law enforcement and PWUD, and an increase in public understanding of substance use as a public health issue and awareness of decriminalization and its role in reducing stigma [8] Many of these goals will also rely on complementary and concurrent system changes such as addressing social determinants of health (e.g., housing, poverty, racism, etc.) and increasing resources for community health and social services. However, many of these goals have been contested as being logically linked to the decriminalization initiative *per se*. For instance, many advocates and PWUD have pointed out the tenuous linkage between decriminalization and health outcomes, including specifically in relation to increased access to and engagement with health and social services, the reduction in illicit drug poisoning deaths, and the ability to reduce the stigma associated with drug use [36]. Additionally, concerns have been raised regarding the possession threshold in particular, which many individuals perceive to be too low or arbitrary in nature [37]. Given the breadth of these goals, as well as their contested nature, it will be imperative to examine and evaluate each of their individual and combined contributions to be able to determine the overall success of this policy and to confirm whether the expected goals of the policy are being realized.

To achieve this objective, the Ontario Node of the Canadian Research Initiative in Substance Matters (CRISM; a national research network focused on conducting research and translating it into evidence-based interventions for substance use), has received a 5-year grant (2022–2027) from the Canadian Institutes of Health Research to evaluate BC's decriminalization initiative. The evaluation will use implementation science research methodologies throughout its five-year duration and will encompass a comprehensive range of research approaches including quantitative, qualitative, and mixed-methods studies, as well as public opinion surveys and an economic evaluation. This extensive research, which will include several sub-studies, will focus on key areas that include PWUD, police and the criminal justice system, the general public, and the healthcare system. The evaluation will assess the effectiveness of BC’s decriminalization initiative in these specific domains as well as its overall impact, aligning with the initiative’s stated goals. The following protocol provides a detailed outline of the evaluation’s design, as well as the specific objectives, methodologies, and planned analyses for each of the individual sub-studies that comprise the evaluation [38, 39].

## Methods

The current proposed evaluation is multifaceted and encompasses several sub-study designs that are observational in nature. The evaluation’s primary objectives are to generate evidence on the public health and economic impacts of BC’s decriminalization initiative by engaging stakeholder groups, fostering cross-disciplinary collaboration with decision-makers and knowledge users, considering the local context and other factors such as biological and social determinants of health, identifying considerations for further evaluation activities, and advancing evidence-based practices through knowledge dissemination activities.

### Stakeholder engagement

**Fig. 1:**
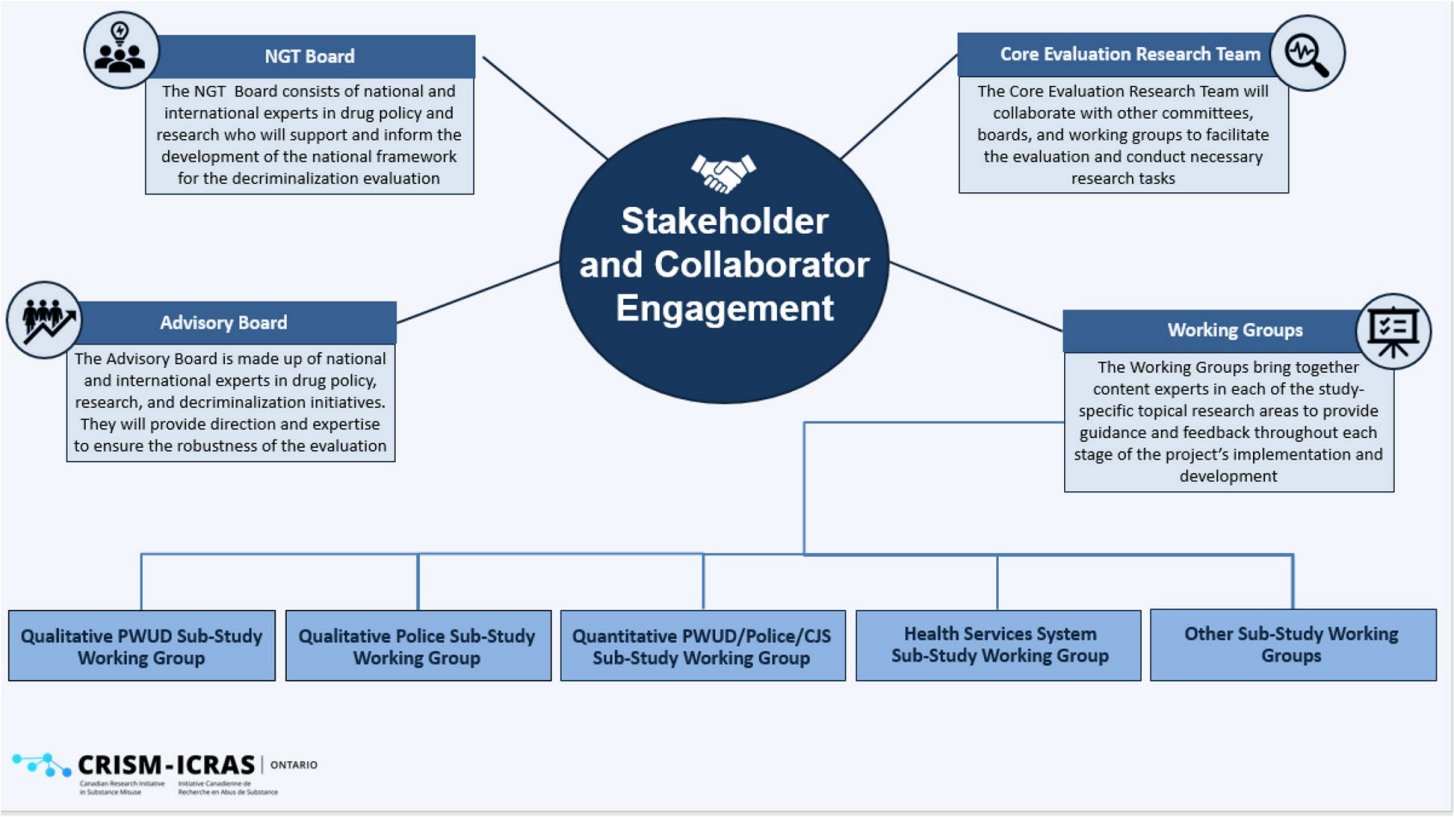
Stakeholder collaboration and engagement

To achieve these objectives, the evaluation has been designed with a strong focus on stakeholder engagement and collaboration [40]. Within the evaluation framework, four distinct groups play integral roles, each contributing uniquely to the research process: 1) the core evaluation research team; 2) the nominal group technique board; 3) the advisory board; and 4) the sub-studies’ working groups (see Fig. 1 for stakeholder engagement diagram). The core evaluation research team encompasses multi-disciplinary researchers and knowledge users from diverse backgrounds and broad expertise. This group is responsible for drafting the initial evaluation design and supporting and facilitating the implementation of the evaluation. These members also have roles within each of the individual sub-studies that comprise the overall evaluation. The nominal group technique board was a time-limited group consisting of world-renowned international and national experts in drug policy research and PWUD. The nominal group technique board members were brought together to undertake a nominal group technique process which incorporated four steps: 1) the core evaluation research team circulated the initial draft evaluation design to the nominal group technique board members and they each weighed in and voted on the design, 2) the core evaluation research team then incorporated the nominal group technique board members’ feedback and revised the evaluation design, 3) the core evaluation research team presented the revised design to the nominal group technique board members during a four-hour virtual meeting that included group discussions and final voting for consensus on the design, and 4) the core evaluation research team finalized the evaluation design and circulated it back to all nominal group technique board members. In addition, an advisory board was also established. The advisory board is similarly comprised of national and international experts in the fields of drug policy, research, and decriminalization initiatives. Notably, some members from the nominal group technique board also serve on the advisory board, facilitating the exchange and sharing of insights and perspectives in different capacities. This group will convene quarterly throughout each year of the five-year evaluation. Lastly, each sub-study will have a working group consisting of relevant topical experts including: academics, researchers, community health providers/practitioners, policy makers, and PWUD. These individuals will provide guidance and feedback throughout each stage of the development and implementation of individual sub-projects and will contribute to sub-study knowledge translation activities.

### Logic model and evaluation design

**Fig. 2:**
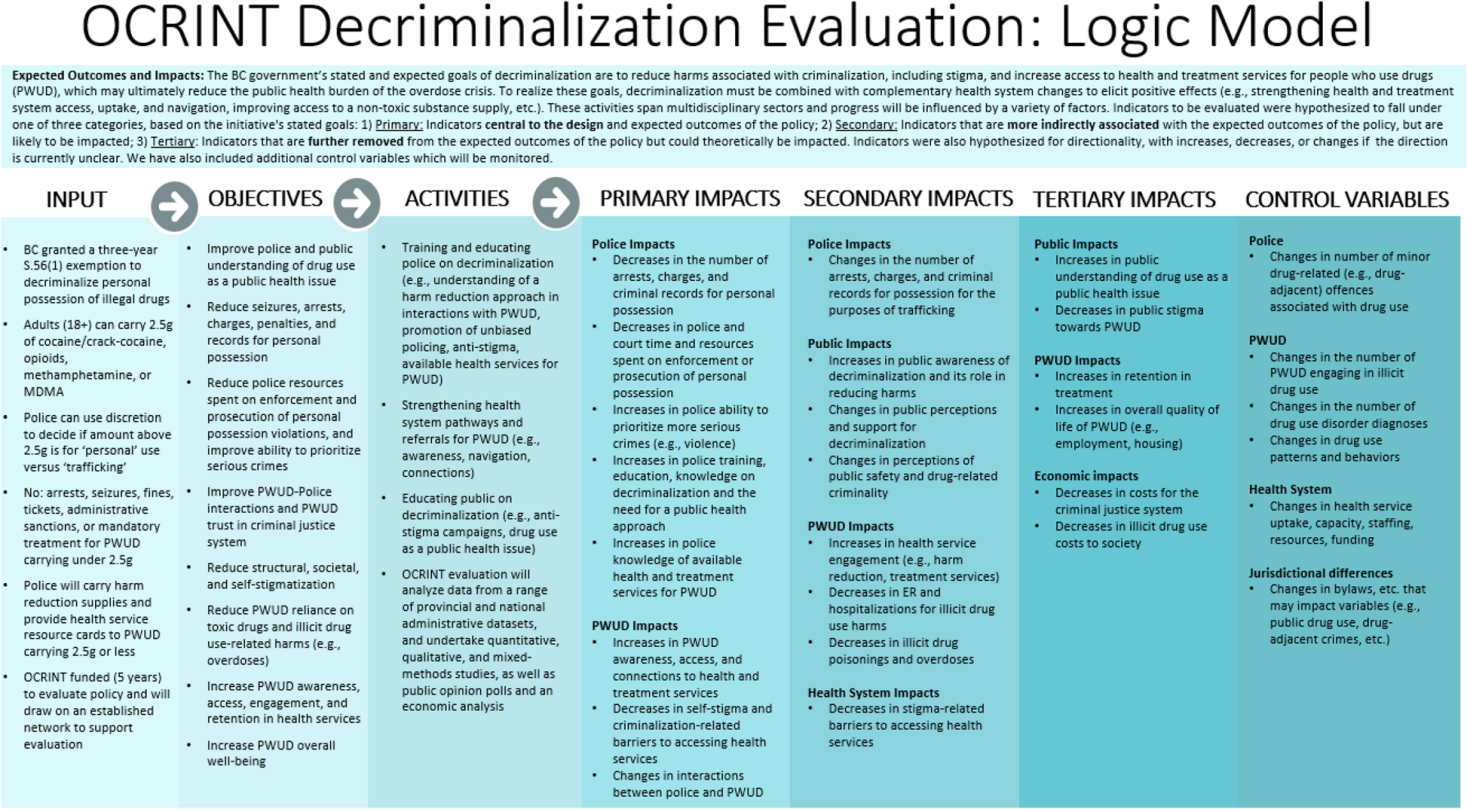
Decriminalization evaluation logic model

Informed by the grant’s identified key areas of research and objectives, as well as feedback from the nominal group technique board, the final evaluation design and corresponding logic model were developed. The logic model includes key inputs, objectives, and activities of the decriminalization initiative, as well as hypothesized impacts of decriminalization, primarily based on the BC government’s stated and expected goals. These are further broken down into three categories: 1) Primary impacts: indicators closely aligned with the policy’s expected outcomes, 2) Secondary impacts: indicators of significance that may not be directly linked to the expected outcomes of the policy but are of importance and are likely to be influenced by the policy, and 3) Tertiary impacts: indicators situated further from the anticipated outcomes of the policy but which could theoretically be influenced. The logic model also includes a list of relevant indicators which will be used as control variables (see Fig. 2 for logic model).

**Fig. 3:**
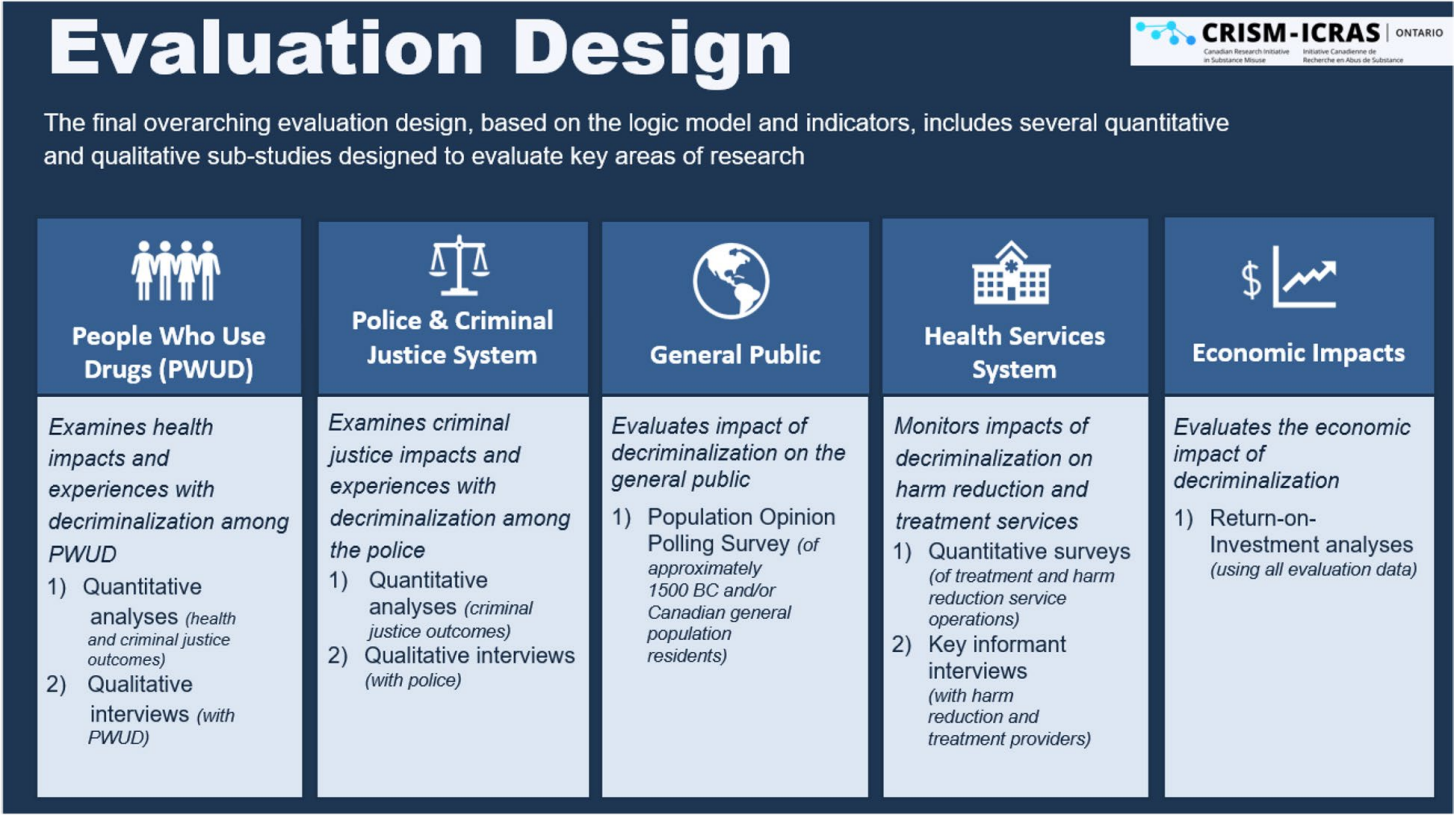
Overarching evaluation design and individual sub-studies

The final evaluation design consists of eight interconnected sub-studies, each designed to explore specific topical areas with the broader scope of our research focus. These sub-studies collectively address five distinct thematic domains: 1) PWUD, 2) police and the criminal justice system, 3) the general public, 4) the health services system, and 5) the economic impacts. All data will be integrated where applicable (e.g., qualitative data will supplement quantitative data and vice versa) to increase the robustness of our findings (see Fig. 3 for evaluation study design).

### Individual sub-studies

#### People Who Use Drugs (PWUD)

To assess the impacts of decriminalization on PWUD, we will undertake two distinct sub-studies: a quantitative and a qualitative study.

*Quantitative PWUD sub-study*

Goals and study design:

The objective of the quantitative PWUD sub-study is to examine the impacts of decriminalization on the health and substance use-related outcomes of PWUD. We will leverage existing population-based surveys and administrative databases. Outcomes will be assessed using monthly counts with data being collected retrospectively from 2013–2022 (where available), and prospectively from 2023–2027. Analogous outcomes from other provinces and territories that have not implemented decriminalization will also be collected to serve as a comparison (where possible depending on data availability and comparability).

For outcomes where comparison data is not available from other provinces and territories, an interrupted time series framework will be operationalized. These outcome domains include prescriptions of opioid agonist treatment (OAT) medications, overdose prevention services utilization, paramedic attended illicit drug overdoses, and deaths due to illicit drug overdose. For outcomes with comparison data, a difference-in-difference framework will be operationalized. These outcome domains include hospitalizations due to drug use and load per capita of drugs in wastewater, which will be utilized as a proxy measure for substance use.

Data acquisition will involve collaborations with three key organizations: The Canadian Institutes for Health Information, Statistics Canada, and the British Columbia Centre for Disease Control. See Appendix A for detailed information pertaining to each outcome domains’ primary outcomes, data collection period, database name, source, coverage, and description.

Statistical power:

All outcome data will be aggregated through the population-based surveys and administrative databases which represent censuses of the population. Power calculations performed in other types of statistical analyses using the G*Power Software are not suitable in time series analyses [41]. However, experts recommend 50 to 100 observations as a rule of thumb [42–44], as well as two years of monthly data to enable adjustment for seasonality [45]. The pre-decriminalization of possession of illicit drugs period will include approximately 120 observations, and the post-decriminalization of possession of illicit drugs period will include approximately 60 observations, given the proposed timelines of the data collection. As such, there will be sufficient statistical power to conduct the analyses and sufficient observations to account for seasonality in the analyses.

Proposed analyses:

All data will be sex-specific (male, female) and age-specific (0–18 years, 19–39 years, 40–59 years, ≥ 60 years), where possible. These data will be used to compute sex-specific, age-standardized rates per 100,000 population.

For outcomes where comparison data are not available from other provinces and territories, we will implement an interrupted time series framework using generalized additive mixed models with a suitable distribution. The beginning of the intervention will be coded as February 2023. Seasonality will be accounted for in the analyses by the inclusion of a smoothing spline. The models will test for changes in both the level and slope (including non-linear changes) of the outcomes. Order of the auto-regressive and moving-average series will be determined through the auto.arima function of the forecast package in R and confirmed by the visual examination of the respective plots of the autocorrelation function and partial autocorrelation function. They will be included in the final model if their inclusion results in a better model fit as indicated by a higher value of R-Square or lower values of Bayesian Information Criterion or Akaike Information Criterion. The Shapiro–Wilk test and Q-Q plots will be used to assess residual normality, and residual plots against the linear predicted values will be examined to determine stationarity.

For outcomes where comparison data are available from other provinces and territories, we will employ a difference-in-difference framework using generalized estimating equations for repeated measures with a suitable distribution. The models will include coefficients for time (pre-implementation of policy vs. post-implementation of policy), provincial and territorial policy implementation (other provinces and territories vs. BC), and the interaction of time by provincial and territorial policy implementation. The time period pre-implementation of the policy in other provinces and territories and BC will be compared through visual examinations. If violations of the assumptions of the model are observed, other statistical methodologies will be considered (e.g. comparative interruptive time series, regression discontinuity), as appropriate. Importantly, outcomes pertaining to load per capita of drugs in wastewater will not be analyzed using a difference-in-difference framework, given the limited data from the pre-decriminalization period available for the analyses. These outcomes will be descriptively characterized by jurisdiction using Joint Point Regression.

Although interrupted time series modeling and difference-in-difference modeling are quasi-experimental methodologies focused on causal inference that are commonly used in evaluation of policies, to provide additional control of bias, two confounders will be controlled for in all analyses. First, given the established relationship between economic circumstances and drug use, all analyses will include a covariate capturing the unemployment rate, which will be sourced from Statistics Canada. Second, due to the potential impact of COVID-19 restrictions (e.g., lockdowns and masking) on illicit drug use and illicit drug overdose deaths [32, 46], all analyses will include a covariate reflecting the proportion of days in a month spent under masking or lockdown restrictions if relevant.

For outcome domains where comparison data are not available from other provinces and territories, related data (e.g., pertaining to prescriptions of opioid agonist treatment medications, overdose prevention services utilization, paramedic-attended illicit drug overdoses and deaths due to illicit drug overdoses) will be sourced and characterized using Joint Point Regression through public dashboards from Alberta [47] and Ontario [48, 49] (where available) to contextualize the trends observed in BC.

Analyses will commence once a full year of data for 2023 are available and will be conducted on an annual basis as new data become available. The statistical significance threshold will be set at *p* < 0.05. Missing data are not anticipated as the population-based surveys and administrative databases represent censuses of the population. All analyses will be conducted using SAS, R, and Joint Point Regression Software.

*Qualitative PWUD sub-study*

Goals and study design:

To complement the quantitative analyses, we will conduct four annual rounds of qualitative interviews with approximately 100 PWUD a year from across BC to gain an in-depth understanding of the impacts of the decriminalization policy among PWUD. This initiative will begin in late 2023, with each assessment seeking to recruit new participants. This approach aims to ensure a broad sample of PWUD with diverse substance use patterns, lifestyles, and locations. Specifically, we will seek to recruit participants from the five BC Health Authorities (i.e., Interior, Fraser, Vancouver Coastal, Vancouver Island, and Northern) using quota sampling.

Recruitment will leverage the Ontario CRISM Node’s established connections with relevant health organizations (e.g., the British Columbia Centre for Disease Control, British Columbia Centre for Substance Use), and community-based drug user advocacy groups throughout the province (e.g., the Peer Engagement and Evaluation Project, the Vancouver Area Network of Drug Users, the Canadian Association of People Who Use Drugs, Kelowna Area Network of Drug Users, Nanaimo Area Network of Drug Users, Coalition of Substance Users of the North, Rural Empowered Drug Users Network, and the Coalition of Peers Dismantling the Drug War). Additionally, we will circulate our study posters and flyers on social media networks and within relevant organizations. Snowball sampling techniques will be employed to reach PWUD less connected to health and harm reduction services or peer advocacy groups. Recruitment and interviews will continue until data saturation is achieved within each major health authority/delivery area. If issues of over- or under-representation of socio-demographics (e.g., age, sex/gender, substance use patterns, geographic location) arise, targeted recruitment efforts will be undertaken. This comprehensive recruitment strategy will help ensure that we recruit diverse PWUD, including those facing marginalization or vulnerability (e.g., individuals experiencing homelessness), those who reside in rural and remote communities, and both high- and low-frequency substance users.

Interested individuals will contact our toll-free study hotline or email the study contact to undergo eligibility screening. Eligibility criteria include individuals aged 18 + residing in BC since before decriminalization and using illicit substances at least three times weekly. The screening will also entail a brief socio-demographic survey to capture relevant data (e.g., age, race, ethnicity, sex, gender, sexual orientation, employment and housing status, geographic location including rurality, substance use, and prior involvement in decriminalization-related research). The eligibility screener will be administered using Research Electronic Data Capture (REDCap) online survey software (a secure web application for building and managing online surveys and databases) [50]. All participants will provide informed consent prior to the interview. Qualitative interviews, lasting about 45 min, will be conducted virtually (e.g., through Webex/Zoom platforms) or over the telephone, and will be audio recorded. Participants will receive a $50.00 cash honorarium for participating via E-transfer (or Moneygram if they do not have a bank account), in line with standard remuneration policies for PWUD.

The interview guides will be collaboratively developed with the sub-study’s working group. Questions will focus on the impact of decriminalization on participants’ lives, perceptions of the policy and its impacts, experiences with police interactions related to drug use, changes in substance use patterns and behaviours, stigmatization, health and social service utilization, and impacts on overall health and social well-being (see Appendix B for a sample interview guide).

Proposed analyses:

Quantitative socio-demographic data will be analyzed using descriptive statistics to characterize the sample and provide additional contextual insights to complement the qualitative data.

All audio recordings will be transcribed by a third party transcription company and transcripts will be imported into a qualitative data management software system (NVivo version 12) [51] for analysis. We will develop an initial coding framework based on our research questions and reviewing and coding initial select transcripts. The preliminary coding framework will be developed with input from all members of the interview team after discussions and debriefing, supported by study notes and interview memos. Analyses will be thematic and iterative, primarily following Braun and Clarke’s (2017) contemporary approach to thematic analysis and related methods [52]; however, specific analytical or theoretical lenses, frameworks, and approaches to coding may differ depending on the specific outputs of interest. Analyses will largely entail familiarizing ourselves with the data by reading and coding the transcripts in several rounds, initially coding larger themes, working our way through the data with each coding pass to highlight additional sub-themes. Coding and analyses will be done in collaboration with the core research team and working group members who will meet regularly during the coding process. Results will be informed by major themes derived from the data.

#### Police and the criminal justice system

Similar to the PWUD sub-study design, we will conduct two separate sub-studies to evaluate the effects of decriminalization on the police and the criminal justice system. These sub-studies comprise both qualitative and quantitative research methods.

*Quantitative police and criminal justice system sub-study*

Goals and study design:

The objective of the quantitative police and criminal justice system sub-study is to examine the impact of decriminalization on criminal justice-related outcomes. We will analyze three administrative databases available from Statistics Canada: the Uniform Crime Reporting Survey, the Integrated Criminal Court Survey, and the Canadian Correctional Services Survey. The outcome domains that will be analyzed include police-reported illicit drug-related offenses, illicit drug-related criminal charges, and number of persons incarcerated for illicit drug-related offenses. These outcomes will be measured using monthly counts with data being collected retrospectively from 2013–2022 (where available), and prospectively from 2023–2027. See Appendix C for detailed information pertaining to each outcome domains’ primary outcomes, data collection period, database name, coverage, and description.

Proposed analyses:

As the data are available for all provinces and territories, a difference-in-difference framework using generalized estimating equations for repeated measures with a suitable distribution will be implemented (see proposed analyses section above in the PWUD quantitative methods section for further details).

*Qualitative police and criminal justice system sub-study*

Goals and study design:

Similar to the PWUD qualitative study outlined above, we will conduct annual qualitative interviews with approximately 30–40 police officers and/or criminal justice system representatives (e.g., judges, prosecutors/lawyers, police union members, probation/parole officers, drug court workers) per year over five years (starting in late 2023) to understand the impacts of decriminalization on the police and the criminal justice system. To promote the relevance and reach of this study, we will partner with researchers from Simon Fraser University who have prior experience and relationships in the local context. The study leads will collaborate closely with the core evaluation research team to ensure the data collection and findings are relevant to and inform the national evaluation. We will establish formal service exchange and data sharing agreements to facilitate the collaboration.

Recruitment will occur through several sampling techniques. Researchers will send study information to known contacts in police and criminal justice system departments as well as other law enforcement administrators to assist in advertising the study and recruiting individuals. Recruitment will focus primarily on those directly impacted by decriminalization in BC (e.g., officers who patrol the street and/or regularly interact with PWUD, units/police divisions that focus on drug crimes, such as drug and trafficking units, prosecutors who lay drug-related charges, probation/parole officers for individuals charged with drug-related offences). We aim to capture data from different police departments across BC (e.g., municipal police departments, Royal Canadian Mounted Police) as well as the criminal justice system (e.g., courts).

Interested individuals will email the research coordinator to express interest in participating and undergo eligibility screening. The eligibility screening will involve confirming that the participant is an active police officer or criminal justice system employee working in the province of BC, and will also entail a brief socio-demographic survey (e.g., name of department, age, race, ethnicity, sex, gender, rank, years in their current jurisdiction, geographic region, and prior decriminalization research involvement). The sociodemographic information will be interviewer-administered and captured in a separate password-protected file.

All participants will provide informed consent prior to the interview. All qualitative interviews will take place either virtually (i.e., over Zoom) or over the telephone, will last approximately 45–60 min, and will be audio recorded. All participants will be offered $30.00 honoraria for their time and expertise via E-transfer. The recordings will be transcribed verbatim and any identifying information such as names and places will be anonymized. We will continue to interview participants each year until we reach data saturation.

The interview guide will be developed in collaboration with working group members. Interview questions will focus on the impact of decriminalization on the police and criminal justice system employee’s day-to-day work, interactions with PWUD, as well as their perceptions of the policy and its impacts. Specifically, we will ask about decriminalization training and education, preparedness for the decriminalization roll-out, interactions with PWUD (including in relation to discretion, drug seizure, charges laid, plea deals, diversion), knowledge and availability of health services for PWUD, experiences with distributing health resource cards, public consumption and municipal/provincial bylaws related to public consumption, involvement in the decriminalization policy planning process, resources spent on enforcement or implementation of decriminalization-related laws and changes, and operational impacts including any changes to police and criminal justice system priorities. See Appendix D for interview guide.

Proposed analyses:

Descriptive statistics will be applied to the quantitative socio-demographic data, which will be used to describe and contextualize the sample. Qualitative analyses will follow the same procedures as outlined in the qualitative PWUD proposed analyses section above.

#### General public

Goals and study design:

To evaluate the impact of decriminalization on the public, we will conduct an annual public opinion poll among the adult general public residing in BC to elicit their perspectives on the decriminalization of illicit drugs. This project will be administered by a third-party polling company, Ipsos, which will handle recruitment, survey fielding, analysis, and will share the raw data and final reports with the research team. This survey will be conducted each year for four consecutive years starting in early 2024, allowing us to track changing public perceptions of drug decriminalization over time.

For recruitment, Ipsos will utilize its existing panels of potential participants who agree to participate in surveys for rewards or incentives (i.e., panelists earn points which can be redeemed through Amazon gift cards, PayPal, etc.). Ipsos has its own supply of survey respondents through its globally managed Ipsos iSay panels. In addition, Ipsos partners with many different types of external suppliers to source samples when needed to fulfil project requirements. In Canada 60% of the volume is recruited through social media, 36% through affiliate networks and media agencies, and 4% through self-recruitment and referral.

To reflect the general public’s views, we will recruit and weight participants based on age, sex, gender, ethnicity, housing status, and level of education. Ipsos will employ quotas and proprietary algorithms to match official census statistics, closing the survey for specific demographic groups once their target quota is met. Each survey will include approximately 1,200-1,500 participants.

Eligible participants will be 18 years of age or older, reside in BC, speak and comprehend English, and have access to a tablet or computer. Ipsos will apply exclusion criteria to maintain panelist engagement and eliminate bias from overusing the same respondents. Ipsos panelists will provide their socio-demographic information (age, sex, location) at the beginning of the survey and if they do not meet the above-mentioned eligibility criteria, they will be screened out of the study. These rules are based on their panel management expertise and are aimed at eliminating the bias resulting from overusing the same respondents, while maintaining panelist engagement.

Panelists will receive standardized email invitations to participate and can opt-in to take the survey. They will also be able to access surveys through the panelist website or app dashboard. Ipsos does not reveal the topic of the survey to limit bias. Respondents will receive a specific informed consent text outlining the nature of the study, participant rights, and contact information if they have questions about the study. Respondents will be able to withdraw from the study by emailing Ipsos.

Survey questions will be developed by the core evaluation research team and will focus on public awareness of decriminalization, support or opposition to decriminalization, perceptions and experiences of public safety and drug-related criminality, public understanding of substance use as a public health issue, and public stigma towards PWUD. See Appendix E for sample survey questions.

Proposed analyses:

Ipsos will provide frequencies of survey responses and cross-tabs of each question by demographics such as age, gender, region, education and household income. Data will be weighted to approximate the demographics of adult BC residents. In addition, both Ipsos and the research team will review the open-ended responses and code them into themes to quantify this data. The aggregate and de-identified data, along with reports and PowerPoint presentations, will be provided by Ipsos to the core evaluation research team. The same statistical methods will be applied to each survey iteration over the five-year study, with inclusion of additional statistical tests of association to detect changes in responses over time.

#### Health services system

Goals and study design:

To examine the impact of decriminalization on the health services system, we will conduct a longitudinal mixed-methods study involving an annual quantitative survey administered to individuals who work at harm reduction and opioid agonist treatment (OAT) services. Specifically, we will routinely capture and collect province-wide data pertaining to harm reduction and OAT service operations. Subsequently, we will conduct key informant qualitative interviews to provide additional context and nuance to the quantitative data. The study will span four years, commencing in early 2024.

Participants will be recruited from OAT clinics and various harm reduction services across the five BC health authorities. Eligible harm reduction services include those with a *primary purpose* of providing harm reduction services including Supervised Consumption Sites, Overdose Prevention Services, Rapid Access Addiction Medicine/Rapid Access to Addiction Care clinics, Mental Health and Substance Use sites, as well as low-barrier harm reduction sites that offer safe supplies, drug checking, addiction treatment, and safe needle disposal. Shelters and/or supportive/temporary housing settings or mobile units that are registered as overdose prevention sites will also be included. Services where the primary purpose is not harm reduction will be excluded. Broader community health centers/clinics, health units, and/or pharmacies that distribute harm reduction supplies will be excluded from our study, as will sites that specifically service youth and those that opened post-decriminalization. Eligible OAT services will include standalone OAT clinics, Rapid Access Addiction Medicine/Rapid Access to Addiction Care clinics, Mental Health and Substance Use sites that have integrated OAT clinics, or OAT clinics that are integrated within harm reduction sites. Broader community health centers/clinics, health units, pharmacies that distribute OAT medications, general practitioners/physicians who prescribe OAT through primary or emergency care settings, and sites catering specifically to youth (i.e., sites that only serve those under 18 or have a youth-specific focus) will be excluded from our study. OAT sites that opened after the decriminalization policy will also be excluded.

To identify eligible services, we will initially draw on a well-established British Columbia Center for Disease Control website (i.e., Toward the Heart) [53] which hosts an interactive map and corresponding list of established harm reduction programs across BC, as well as a publicly available list of clinics accepting new OAT patients developed by the British Columbia Centre on Substance Use [54]. Using these resources, we will develop an initial list of potentially eligible services which we will circulate and share within our networks (e.g., the British Columbia Center for Disease Control Harm Reduction Coordinators, the Decriminalization Leads of each of the five Health Authorities in BC, and peer advocacy groups), as well as with the sub-studies’ working group to confirm service eligibility and identify any missed services or those that do not fit our eligibility criteria. We will also rely on these networks and the working group to help identify a key contact within each site who would be most suitable to complete the survey on behalf of the organization (e.g., program manager, director). For any services on the list for which we are not able to pre-identify a key contact, we will send general solicitation emails describing the study and request that the service identify the most suitable person to participate and complete the survey on the organization’s behalf. Survey respondents must have detailed knowledge of the service’s operations (e.g., know clientele specifics, referral pathways, engagement, funding sources) and be able to speak to any changes that have occurred since decriminalization. We anticipate that the survey will be sent to approximately 110 harm reduction organizations/sites and 75 OAT organizations/sites each year, with an anticipated response rate of between 50–70%, in line with average response rates for health care surveys [55]. Once an organizational contact person has been identified, survey dissemination will be strategically coordinated. Our Decriminalization Lead contacts within the five regional health authorities and the British Columbia Centre for Disease Control Harm Reduction Coordinators will forward emails including information on the purpose of the survey, site eligibility information, and a link to the consent form and survey to the contacts they identify. For sites where we independently identify contacts, we will send the survey invitation link directly to them by inputting their email into the REDCap platform and enabling automatic email invitations. We will set the length of the survey fielding at approximately two months*,* sending the initial invitation followed by two reminder emails approximately once every two weeks.

We will create two distinct surveys: one for harm reduction services and one for OAT services. Sites that offer both harm reduction and OAT services will be given the option to complete both surveys. Surveys will be created in collaboration with our working group and will include questions that generally focus on the following aspects: service capacity, provider/role information (e.g., type of role, how long they have worked there), waitlists, eligibility criteria, types of services offered, service utilization/uptake, treatment retention, resources (personnel, infrastructure, and funding), referral pathways, adherence to guidelines, clientele specifics (demographics, substance use profiles), and changes in these outcomes since decriminalization. See Appendix F for sample surveys. Upon completion of the survey, respondents will receive a $25.00 Amazon e-gift card for participating and will have the option to provide their email address if they are interested in participating in a qualitative key informant follow-up interview.

Similar to our surveys, for the follow-up interviews we will develop two separate semi-structured qualitative interview guides: one for harm reduction site participants and one for OAT site participants. The interview guides will be developed in collaboration with working group members, including PWUD and service providers, and will be based on the survey responses which will allow us to gain more in-depth information on aspects from our survey results. As we will use our quantitative survey responses to inform the qualitative survey interview guides each year. We anticipate that the qualitative interview data collection procedures will be staggered, occurring a few months after survey data collection each year. We anticipate conducing approximately 10–15 key informant interviews per service type per year. Interested individuals will be contacted by a member of the research team to arrange a time to conduct the interview. Semi-structured qualitative interviews will be one-on-one, audio-recorded, conducted over the phone or videoconferencing platforms, and will last approximately 30–45 min. Participants will be provided with a $50 Amazon e-gift card for their participation in these interviews.

Each year before the survey roll-out, we will re-confirm the eligibility of services (e.g., add any additional eligible services or remove ones that no longer meet eligibility or have closed) and will also re-confirm participant contact information in the same manner as our initial recruitment procedures.

Proposed analyses:

For data analysis, quantitative survey data will be exported and descriptive statistics will be computed. Qualitative analyses will follow the same procedures as outlined in the qualitative PWUD proposed analyses section above.

#### Economic impacts

Goals and study design:

In the final year of the evaluation, we will undertake a Return on Investment analysis which will seek to compare the costs of decriminalization and its potential benefits, both expressed in monetary terms. A return on investment analysis is an economic measure used to understand how much economic benefit is derived from a policy compared to its cost [56]. These analyses are increasingly being used to evaluate healthcare policies [57, 58]. Given the wide-reaching implications of the decriminalization policy, including the potential impacts on several important sectors such as the criminal justice and health services system, it will be imperative to understand the full benefits and costs of this policy. As such, the current proposed return on investment will quantify all costs of decriminalization and put it into relation with potential savings, for example, resulting from reductions in arrests and convictions for possession of illicit substances. All costs and benefits will be assessed from a societal perspective and the timeframe will be four years. Costs will be derived in line with current practices for cost studies on drug use including the World Health Organization’s International Guidelines for Estimating the Costs of Substance Use [59], and the Canadian Centre for Substance Use and Addiction’s Centre on Substance Use Costs and Harms website and associated data visualization tool [60]. Additional costs will be assessed using questionnaires to the institutions where such costs were incurred. Benefits will rely on the other data collected throughout the evaluation, which will determine the net difference in costs incurred causally linked to decriminalization.

Proposed analyses:

Specifically, the return on investment will incorporate four phases: 1) estimate the economic burden, 2) estimate the cost of the intervention, 3) estimate the impact of the intervention, and 4) quantify the return on investment of the intervention, using the classic formula: Return on investment = Net savings of the intervention/cost of the intervention.

## Discussion

The protocol described above outlines a multifaceted evaluation of BC’s decriminalization of illicit drugs policy. This evaluation encompasses eight interrelated sub-studies, each employing qualitative, quantitative, or mixed-methods approaches within five distinct topical areas. Collectively, these sub-studies provide a comprehensive and robust framework to assess and evaluate the overall effectiveness of the decriminalization policy and to allow comprehension of the full spectrum of its impacts.

### Potential to explore additional research activities

To enhance the validity, accuracy, and robustness of our evaluation and the data collected within each sub-study, we will also explore additional research activities as required and appropriate as the evaluation unfolds. For instance, one caveat of the decriminalization policy is that although it is universally mandated across the province, how the policy is applied and enforced will vary by jurisdiction and specific police forces. It will also be dependent on resources allocated towards specific services within jurisdictions and how they are able to respond to the policy. It will therefore be important to conduct jurisdictional-specific analyses within and across our sub-studies to examine potential differences in the application of the decriminalization policy. Additionally, it will be important to triangulate our findings across all sub-studies, comparing and contrasting results to examine potential associations and to see whether and/or where data significantly differ. Furthermore, we will conduct member checking exercises wherein we will provide summaries of our work to key stakeholder groups to ensure that our results resonate and align with the experiences of these individuals, and will examine potential ‘negative cases’ (i.e., viewpoints of respondents that differ from the findings) to discern whether and how these individuals’ experiences refute our findings.

### Challenges & limitations

Given the breadth of this evaluation, there are several experienced and anticipated challenges. A primary challenge encountered from the onset of the decriminalization evaluation has been related to multiple concurrent initiatives exploring its impact. Specifically, there is considerable overlap between our evaluation and other evaluation studies, including the internal evaluation being led by the Province of BC and funded by Health Canada. This occurrence has led to the overburdening of certain participant groups such as PWUD and health system service providers who have been approached to participate in multiple studies. Additionally, we have encountered difficulties identifying potential collaborators who are not already engaged in the other evaluations, creating conflicts of interest. Considering our study is an independent evaluation, it is important that we avoid any overlap with the provincial project to ensure the distinctiveness and credibility of both evaluations. Consequently, one of our strategies has been to carefully screen potential participants to avoid including individuals already involved in similar research. This screening process, incorporated into our participant selection criteria, aims to prevent participant over-sampling, and reduce the strain on specific services and sites. Our goal is to maintain the integrity of our evaluation by ensuring a distinct and representative participant pool, thereby enabling us to accurately assess the decriminalization policy's impact without the confounding effects of concurrent studies.

Another sizeable challenge that we anticipate is data availability. Certain desired data, such as drug seizures, emergency department visits, and utilization of addiction treatment services are unavailable, limiting the overall scope of our analysis. As such, our evaluation is restricted to available data, resulting in the omission of some outcomes and associated analyses. Moreover, for the data that are available, there are inherent time lags between when the data are collected, finalized, and available. This may result in delayed analyses and generation of findings, and will necessitate a nuanced approach to data interpretation that must consider potential factors that may have impacted the data during the lag time.

External factors, notably the COVID-19 pandemic and other policy initiatives may pose potential limitations. COVID-19 has drastically altered the healthcare system and societal behaviours [32, 61]. Similarly, medical safe supply programs, which refer to the provision of prescribed medications to PWUD as a safer alternative to the toxic illegal drug supply may influence our results [62]. Additionally, as of May, 2024, the BC government re-criminalized public consumption and use of drugs, making it illegal to consume or possess any amount of drugs in public, limiting the decriminalization initiative to private residences or places where people are legally sheltering, or to healthcare settings that have been granted an exemption under the *Controlled Drugs and Substances Act* (e.g., supervised consumption sites) [63]. This policy amendment will undoubtedly result in potential confounding effects on the outcomes of interest within this evaluation. To address and account for these potential impacts, we will adjust our statistical models within our quantitative sub-studies, and will include specific questions on the impact of re-criminalization within our qualitative and mixed-methods sub-studies.

Furthermore, there are many biases inherent in qualitative and mixed-methods research, including sampling/selection biases, interviewer biases, response biases, and analysis biases, all of which must be acknowledged. We will make every effort to mitigate these types of biases. Mitigation strategies will include employing diverse sampling methods (e.g., expanding snowball-sampling techniques to reach individuals who are less connected to services or have different substance use profiles), practicing reflexivity (i.e., acknowledging our own inherent biases and positionality throughout each stage of our studies), ensuring data saturation, and incorporating peer debriefing and member checking. These measures are integral in mitigating biases and enhancing the validity of our findings.

Lastly, some minor challenges that we have experienced thus far have included time commitments spent on stakeholder engagement, preparing and executing legal agreements with partner institutions, submitting multiple research ethics board applications, and identifying appropriate collaborators. Some potential collaborators have also declined participation or are not interested in participating.

### Knowledge translation

Given that creating and disseminating research evidence is essential in order to effectively convey our findings to different audiences, we have developed a framework for knowledge translation. For each sub-study, at semi-annual or annual intervals depending on the study specifics, we plan to develop a diverse set of relevant knowledge translation materials [64]. These will include semi-annual progress reports and virtual presentations for the funder and relevant stakeholders. The reports will detail ongoing work, interim findings, and research milestones with the intent to keep stakeholders and funders informed about the evaluation's progress. Presentations to the funder will facilitate a direct exchange of information and feedback.

Additionally, we plan to create public-facing knowledge translation materials intended for the general public, summarizing key findings in an accessible, plain-language and reader-friendly format, in both English and French. These reports will be a valuable resource for community members, advocacy groups, and anyone interested in understanding the impacts of decriminalization. This information will be hosted online on our Ontario CRISM Node website [65]. To further enhance dissemination, infographics will also be developed to succinctly present data and insights. These visual tools will be designed to be easily shareable on social media platforms and websites, ensuring that our data and findings reach broader audiences. Webinars will be organized to present our findings and engage with specific stakeholders, such as policymakers, service providers, and researchers. Webinars will provide an interactive platform for discussing research outcomes and their implications. Publishing sub-study specific manuscripts in academic peer-reviewed journals and sharing our research results at academic conferences will also allow us to reach specialized audiences of researchers and experts in related fields, which will provide an opportunity for scholarly exchange. We will also conduct an overall five-year integrated report and academic manuscript outlining the most significant findings from all sub-studies. This integrated report will provide a comprehensive overview of the decriminalization evaluation’s key outcomes and policy implications. For all knowledge translation materials and outputs, we will work closely with our CRISM network and collaborators. This collaboration will help us disseminate our findings effectively, ensuring they reach a wide range of stakeholders and communities.

### Impacts of the research

The outcomes of this research will have important implications, particularly in the sphere of drug policy reform. The impacts are expected to resonate at various levels including municipal, provincial, national, and international. The results from each of the sub-studies will inform policy discussions, with the potential to lead to informed revisions and recommendations regarding the decriminalization of illicit drugs in BC, which can be adopted or adapted in other jurisdictions. The findings gleaned from the evaluation are poised to offer pivotal insights that can shape and refine the discourse on drug policy. By providing robust, evidence-based results and perspectives, our research will serve as a critical resource for policymakers, advocates, and stakeholders in understanding the multifaceted impacts of decriminalization policies.

Specifically, the results from our qualitative and mixed-methods studies can provide an in-depth understanding of the impacts of decriminalization on those most affected by the policy – PWUD, police, and health service providers. By engaging these groups and hearing their voices, the evaluation will contribute to reducing stigma and changing societal attitudes, fostering a more compassionate approach to supporting PWUD. Our economic analysis will also provide key information regarding the costs associated with decriminalization and any return on investment, which will be relevant for public spending in relation to healthcare and the criminal justice system costs.

Moreover, the work will foster positive engagement and collaboration with a diverse array of stakeholders which extends to communities, community organizations, advocacy groups, researchers, academics, government officials, and PWUD both nationally and internationally. Such an inclusive and collaborative approach will ensure that the perspectives and needs of diverse groups are being considered, leading to more holistic and effective policy outcomes.

## Supplementary Information

Supplementary Material 1: Appendix A. Detailed table providing information on all quantitative health data to be collected, including primary outcomes, data collection period, database name, source, coverage, and description.

Supplementary Material 2: Appendix B. Sample interview guide for the qualitative sub-study with PWUD.

Supplementary Material 3: Appendix C. Detailed table providing information on all quantitative police and criminal justice system data to be collected, including primary outcomes, data collection period, database name, source, coverage, and description.

Supplementary Material 4: Appendix D. Sample interview guide for the qualitative sub-study with police.

Supplementary Material 5: Appendix E. Sample general population opinion polling survey.

Supplementary Material 6: Appendix F. Sample Harm Reduction and OAT service survey.

## Data Availability

No datasets were generated or analysed during the current study.

## Abbreviations

BC: British Columbia
CRISM: Canadian Research Initiative in Substance Matters
OAT: Opioid Agonist Treatment
PWUD: People who use Drugs
REDCap: Research Electronic Data Capture (survey software)
