# Supplementary material for: The decriminalization of illicit drugs in British Columbia: a national evaluation protocol
Source: BMC Public Health. 2024 Oct 18;24:2879. doi: 10.1186/s12889-024-20336-9 (PMC11490149; doi:10.1186/s12889-024-20336-9)
Supplement: Supplementary file 1 — Supplementary Material 1: Appendix A. Detailed table providing information on all quantitative health data to be collected, including primary outcomes, data collection period, database name, source, coverage, and description. [file 12889_2024_20336_MOESM1_ESM.docx]

# Appendix A: Quantitative PWUD Sub-Study primary outcome, data collection period, database name, coverage, and description of the outcome domains

|  |  |  |  |
| --- | --- | --- | --- |
| **Outcome Domain** | **Data Coverage and Collection Period** | **Primary Outcome** | **Database Name, Source, and Description** |
| Prescriptions of Opioid Agonist Treatment Medications | The first cut of data will be obtained in May 2024, which will include data from 2015-2023. Data will be obtained annually from thereafter until 2027 | 1) Number of clients dispensed opioid agonist treatment medications  2) Number of first-time clients dispensed opioid agonist treatment medications. | PharmaNet (British Columbia); Sourced via BCCDC: An online, real-time system that includes prescriptions for all drugs and medical supplies dispensed from community pharmacies and prescriptions for all drugs dispensed from hospital pharmacies in British Columbia. Importantly, inpatient dispensations are not captured within PharmaNet. |
|  |  |  |  |
| Overdose Prevention Services Utilization | The first cut of data will be obtained in May 2024, which will include data from 2017-2023. Data will be obtained annually from thereafter until 2027 | 1) Visits to overdose prevention services and supervised consumption services. | Overdose Prevention Services Utilization Data (British Columbia); Sourced via BCCDC: Captures service utilization of overdose prevention services or supervised consumption services operated or funded by regional health authorities. Although these services are provided through various services models (e.g. fixed sites, mobile sites, housing-based sites, peer-to-peer services, episodic witnessing), data represent services provided at fixed sites and mobile sites. Data represent most but not all overdose prevention services and supervised prevention services. Data are affected by the public health measures implemented for the coronavirus disease pandemic (COVID-19), weather-related considerations, fluctuations in staffing levels, opening and closing of sites, changes in service models. |
|  |  |  |  |
| Paramedic-Attended Illicit Drug Overdoses (Version involving the analysis of patient care records by the British Columbia Centre for Disease Control) | The first cut of data will be obtained in May 2024, which will include data from 2015-2023. Data will be obtained annually from thereafter until 2027 | 1) Paramedic-attended probable opioid overdose. | British Columbia Emergency Health Services Database (British Columbia); Sourced via BCCDC: Collects information on emergency calls responded to by paramedics, including paramedic impression codes and 9-1-1 dispatch codes. |
|  |  |  |  |
| Hospitalizations due to Drug Use | The first cut of data is estimated to be obtained in the fall of 2024, which will include data from 2013-2023. Data will be obtained annually from thereafter until 2027 | 1) Hospitalizations due to any drug use disorder.  2) Hospitalizations due to any drug overdose. | Hospital Morbidity Database (National [All Provinces]); Sourced via CIHI: Captures administrative, clinical and demographic information on inpatient separations from acute care hospitals, including national discharge statistics from health care institutions by diagnoses and procedures. |
|  |  |  |  |
| Deaths due to Illicit Drug Overdoses | The first cut of data will be obtained in May 2024, which will include data from 2015-2023. Data will be obtained annually from thereafter until 2027 | 1) Deaths due to any illicit drug overdose. | Unregulated Drug Deaths in British Columbia Data (British Columbia); Sourced via BCCDC: Collects information on illicit drug overdose deaths based on toxicological testing performed by the coroner’s service. |
|  |  |  |  |
| Load Per Capita of Drugs in Waste Water | The first cut of data is estimated to be obtained in the fall of 2024, which will include data from 2022-2023. Data will be obtained annually from thereafter until 2027 | 1) Load per capita of amphetamine.  2) Load per capita of cocaine (benzoylecgonine).  3) Load per capita of codeine.  4) Load per capita of ecstasy (MDMA).  5) Load per capita of fentanyl (norfentanyl).  6) Load per capita of methadone.  7) Load per capita of methamphetamine.  8) Load per capita of morphine.  9) Load per capita of oxycodone. | Canadian Wastewater Survey (Select Jurisdictions); Sourced via StatsCan: Collects samples from wastewater treatment plants across multiple municipalities (Metro Vancouver, Edmonton, Prince Albert, Saskatoon, Toronto, Montreal and Halifax) to provide estimates of load per capita (milligrams per one thousand people per day) by drug type and by municipality. |
|  |  |  |  |
