# Supplementary material for: The decriminalization of illicit drugs in British Columbia: a national evaluation protocol
Source: BMC Public Health. 2024 Oct 18;24:2879. doi: 10.1186/s12889-024-20336-9 (PMC11490149; doi:10.1186/s12889-024-20336-9)
Supplement: Supplementary file 2 — Supplementary Material 2: Appendix B. Sample interview guide for the qualitative sub-study with PWUD. [file 12889_2024_20336_MOESM2_ESM.docx]

# Appendix B: Qualitative PWUD Interview Guide Sample

Thank you for taking the time to participate in the study. Before we begin the interview, we would like to remind you that the interview is voluntary and can be stopped at any time or any questions you may not feel comfortable answering can be skipped. The interview will be audio-recorded for transcription purposes. The interview will ask questions related to the decriminalization of drugs and your experiences with the policy. Please confirm that you are okay to continue the interview and for the interview to be audio recorded.

**If no, thank them for their time and conclude the interview.**

**If yes, proceed with the interview.**

Participant ID:___________________________________

We will now go over the open-ended interview questions regarding your experiences and thoughts on decriminalization. You can answer as much or as little as you like.

**INTERVIEW QUESTIONS**

1. Can you please tell me what you know ***about the decriminalization of illicit drugs policy*** in BC?
   - Do you know any specifics of the decriminalization law? If so, can you describe these? If so, do you know where you learned this information?

**For those that do not know much about decriminalization, provide the following brief explanation:**

**In January 2023, BC passed a law to decriminalize the personal possession of a total of 2.5 grams of certain illegal drugs, including opioids, cocaine/crack-cocaine, methamphetamine, and MDMA/Ecstasy, meaning that you can no longer be charged for carrying that amount of drugs on you. This is a pilot initiative, meaning that it is only in place for the next three years, during which time it will be evaluated to see if it is successful. The goals of the policy are to reduce the harms associated with substance use, including stigma and criminalization, as well as to support PWUD in accessing health and social services, ultimately redirecting them away from the criminal justice system.**

1. In general, what are your ***thoughts on this policy?***
   - Do you feel as though it is positive? Why or why not?
   - Negative? Why or why not?
   - Irrelevant/unimportant? Why or why not?
2. Since decriminalization, ***have your drug purchasing patterns changed***? If so, in which ways? If not, why do you think that may be the case? OR do you think your drug purchasing patterns will change as a result of decriminalization?
   - How much do you typically purchase at a time? Are you buying less? More? What is the most you will purchase at one time? Have there been any economic/financial implications related to the drugs that you are purchasing because of the policy? If so, can you describe these?
   - When purchasing and carrying your drugs, do you stay within the cumulative 2.5 gram threshold limit? If so, has this changed since the policy came into effect? What are your thoughts on the 2.5 gram threshold? Too low? Too high? Not relevant/impactful? Cumulative nature? Substances included?
   - How often do you pick up? Has this changed since decriminalization? Are you picking up less? More? If this has changed, why? Do you carry the whole amount on you at one time? Do you pick up for others?
   - Have your interactions with your dealers/suppliers changed at all since decriminalization? If so, in which ways?
   - Since decriminalization, do you feel as though meeting and interacting with your dealer/supplier puts you at a greater or lesser risk for being charged/arrested? Why or why not?
   - Since decriminalization, do you feel that there have been changes to the quality of drugs that you commonly purchase? If so, in which ways have the drugs changed?
   - Are you familiar with safe supply? (provide brief description: regulated/prescribed medications from doctors as an alternative to the illicit drug market)
     - Have you noticed any impacts from safer supply programs in your community?
     - Have you noticed an increase or decrease in the availability of diverted from safe supply medications? (e.g., hydromorphone/Dilaudid)? If so, in which ways? Has the price, quality, or availability of these or other drugs changed?
3. How much do you typically use at a time or in a day? Has this changed since decriminalization? Are you using less? More? If this has changed, why?
   - Has the type of drugs you typically use changed at all?
4. Since decriminalization, do you feel that you are any more or less likely ***to experience drug-related harms or an overdose?*** Why or why not?
   - Do you think decriminalization has the potential to impact (increase/decrease) your risk for drug-related harms or an overdose?
   - Do you feel that decriminalization has the potential to impact the overdose crisis?
   - Since decriminalization, are you any more or less likely to use drugs alone? Why or why not? Do you think decriminalization will impact whether you use drugs alone?
5. How do the police treat PWUD in your community?
   - Since decriminalization, ***has this changed***? If so, please describe the changes you’ve noticed.
   - Before decriminalization, how were the police treating PWUD in your community? Were PWUD concerned about being arrested/charged for personal possession of drugs? Why or why not? Did the police commonly charge people for possession of small amounts of drugs?
   - Has there been a change in how the police treat PWUD in your community since decriminalization? What types of changes have you noticed? Are PWUD concerned about being arrested/charged for personal possession or other drug-related crimes? Do police commonly charge/arrest PWUD for drug-related crimes? Are police more/less present? Do they interact more/less with PWUD?
   - Do you feel as though the way police treat PWUD in your community is any different than how they treat PWUD in other communities? If so, in which ways? Can you describe the differences?
6. Since decriminalization, h***ave your interactions or feelings towards the police changed?*** If so, can you describe how your interactions and/or feelings have changed? If not, why do you think they haven’t changed?
   - Do you think your thoughts or perceptions towards the police will change because of decriminalization?
   - Are you less nervous/scared to be charged/arrested? Why or why not?
   - **(For those who have had police interactions):** Can you describe these experiences? What happened? How did this make you feel? Can you describe the type of interaction? Was it respectful/positive? Aggressive/negative? Have you had encounters with the police for your drug use prior to decriminalization? If so, how do those encounters compare to the encounters after decriminalization? Can you provide an example of the difference in interactions/encounters?
   - Can you describe how the police approached your possession of drugs? (e.g., did they confiscate your drugs? If so, how many grams were you carrying? Did they charge/arrest you for possession, trafficking, or another related charge such as public drug use or loitering, etc.?)
   - **(For those who were fined/charged/arrested):** Can you describe to me what happened to you after the fine/charge/arrest? Do you have to go to jail/court? If so, can you walk me through that experience? What happened? How did this make you feel?
   - Do you feel as though your personal identity (e.g., your sex/gender, ethnicity, housing status, history of incarceration, or other ways in which you identify) has impacted your interactions with police? If so, in which ways? Can you describe this in detail? (e.g., in which ways do you identify? In which ways do you feel these identities impacted your interactions/feelings?)
   - As part of the decriminalization policy, police are expected to provide you with resource cards that identify local harm reduction treatment and services in your area. Have you seen these cards? If yes, what are your thoughts on these cards? Were they useful? Did they facilitate or increase awareness of services?
   - Did the police verbally offer you information or resources on where in your community to go for support?
   - If you received the cards (or verbal instructions), what happened afterwards? Did you follow through and go to the services they suggested? If so how was that experience? If not, how come? Were you interested in the support or services provided? Why or why not? Was there a lack of information on what the service offered or how to get there, etc.? If they didn’t offer you support and services, would you have wanted them to?) How did you feel about receiving the resource card from the police?
   - If not, what do you think of this as part of the policy? Do you think these resource cards will be useful?
   - How do you feel about these referrals or receiving support from the police?
7. Have you ***accessed*** drug use supports and services in your community since decriminalization (e.g., harm reduction, treatment, or other health services)?
   - **(For those who have accessed supports)**: Has your access to or interactions with drug use support changed since decriminalization? In which ways? Increased? Decreased?
   - If so, what do you think has facilitated your access to these supports? How is this different than before decriminalization? Is there anything in particular that has made you want to increase/decrease your access to these supports/services? Are there any barriers to accessing services in your community? Wait times, hours, travel?
   - **(For those who have not accessed supports)**: Can you comment on what it is you think makes you not want to or makes it difficult to access services? Have these changed at all since decriminalization?
   - Do you think the decriminalization policy will increase your willingness to access services or not?
   - Now that drugs are decriminalized, are there services that are available for you to access? Do you feel that there has been an increase in the availability of drug use supports and services? Why or why not?
   - Are there any barriers to accessing services in your community? Wait times, hours, travel?
   - **(For those who are on OAT):** Has decriminalization changed the way you feel about OAT? Is there anything in particular that made you decide to engage in OAT? If so, is this different than before decriminalization?
   - **(For those who are not on OAT):** Has decriminalization changed the way you feel about OAT? Would you consider engaging in it? Is this any different than how you felt before decriminalization? Why or why not?
8. Has decriminalization ***changed your experiences of stigmatization***?
   - Do you feel as though stigma towards you or people who use drugs generally has changed at all since decriminalization? Why or why not?
   - Do you feel as though structural stigma has changed at all since decriminalization? (e.g., barriers to accessing services, being treated poorly or denied care by health care and social service professionals, etc.)? Why or why not?
   - Do you feel as though any self-stigma (e.g., feelings of shame, guilt, etc.) you may feel has changed at all since decriminalization? Why or why not?
   - Do you think that decriminalization will have an eventual impact on stigma down the line or in the future? Why or why not?
9. The BC government has tabled legislation, called Bill 34, around public consumption, which restricts public drug consumption in most public areas. What are your thoughts and feelings about Bill 34 and the restriction of public drug consumption outdoors? Positive/negative

- Do you think this legislation will have a direct impact on your own drug use or the drug use of people you know? Are there specific risks or dangers associated with consuming drugs indoors due to this legislation? More chances of using alone? overdosing?
- Are there particular groups that it will impact more/less, i.e. homeless populations? What are the implications?
- How do you think the new legislation will impact your/PWUD ability to find safe places to use drugs?
- Are there any concerns about seeking help or support if public consumption is prohibited? Are there services you can go to? if you were to commonly use outside, where would you/PWUD go?
- How do you think this legislation will impact the goals of the policy?

1. To your knowledge, a***re there any drug use-related bylaws or regulations in your specific community*** (e.g., laws about public drug consumption) that have been implemented since decriminalization? If so, have these had an impact on you or other PWUD in your community?
2. Has the decriminalization policy ***impacted your life*** in any way? If so, in which ways? If not, why do you think this may be the case?

- Do you feel the decriminalization policy has the potential to impact your life in any way? If so, in which ways? If not, why do you think this may be the case?
  - Since decriminalization, has your quality of life changed at all?
  - If so, in which ways? Employment? Housing? Social situations? Access to support? Reduced drug use/harms? Mental health issues? Please describe any ways in which decriminalization has impacted your quality of life
  - Do you think the decriminalization policy has the potential to improve your quality of life?
  - Do you feel that decriminalization has the potential to impact your life in the future (e.g., next 5**-**10 years?)? If yes, in which ways? If not, how come?

1. Do you have ***any suggestions on ways that the decriminalization policy can be improved?***

- For instance, related to the threshold? Related to the specific drugs included? a need for more connections/referrals to health services? A need for more awareness of the policy? A need for more police training? Need for more services? Safe supply? Longer policy?

1. Do you have any other comments you would like to add regarding your experiences or thoughts on decriminalization?

That concludes our interview, thank you for taking the time to speak with us about your experiences. I will now turn off the recorder.
