# Supplementary material for: The decriminalization of illicit drugs in British Columbia: a national evaluation protocol
Source: BMC Public Health. 2024 Oct 18;24:2879. doi: 10.1186/s12889-024-20336-9 (PMC11490149; doi:10.1186/s12889-024-20336-9)
Supplement: Supplementary file 3 — Supplementary Material 3: Appendix C. Detailed table providing information on all quantitative police and criminal justice system data to be collected, including primary outcomes, data collection period, database name, source, coverage, and description. [file 12889_2024_20336_MOESM3_ESM.docx]

# Appendix C: Quantitative Police and Criminal Justice System sub-study primary outcome, data collection period, database name, coverage, and description of the outcome domains

|  |  |  |  |
| --- | --- | --- | --- |
| **Outcome Domain** | **Data Coverage and Collection Period** | **Primary Outcome** | **Database Name, Source, and Description** |
|  |  |  |  |
| Police-Reported Illicit Drug-Related Offenses | The first cut of data is estimated to be obtained in the fall of 2024, which will include data from 2013-2023. Data will be obtained annually from thereafter until 2027 | 1) Police reported incidents involving illicit drug possession violations of any illicit drug  2) Police-reported incidents involving illicit drug production, trafficking, importation and exportation violations of any illicit drug | Uniform Crime Reporting Survey (National [All Provinces]); Sourced via StatsCan: Collects police-reported crime statistics and is designed to measure the incidence of crime and its characteristics, including offense information for homicides, robberies, breaking and entering, theft (motor vehicle, over and under $5000), stolen goods, fraud, prostitution, gaming and betting, drugs (possession, trafficking, importation/production), weapons and other criminal codes (e.g. bail violations, disturbing the peace, indecent acts, mischief). |
|  |  |  |  |
| Illicit Drugs-Related Criminal Charges | The first cut of data is estimated to be obtained in the fall of 2024, which will include data from 2013-2023. Data will be obtained annually from thereafter until 2027 | 1) Police reported illicit drug possession offenses of any illicit drug where a person was charged  2) Police-reported illicit drug trafficking, importation and exportation and production offenses of any illicit drug where a person was charged  3) Criminal charges related to illicit drug possession  4) Criminal charges related to other illicit drug offenses. | Uniform Crime Reporting Survey (National [All Provinces]); Sourced via StatsCan: Collects police-reported crime statistics and is designed to measure the incidence of crime and its characteristics, including offense information for homicides, robberies, breaking and entering, theft (motor vehicle, over and under $5000), stolen goods, fraud, prostitution, gaming and betting, drugs (possession, trafficking, importation/production), weapons and other criminal codes (e.g. bail violations, disturbing the peace, indecent acts, mischief).  Integrated Criminal Courts Survey (National [All Provinces]); Sourced via StatsCan: Collects information on appearances, charges and cases in youth courts and adult criminal courts in Canada. It represents a census of pending and completed federal statue charges heard in provincial-territorial and superior courts in Canada. Excluded is information from appeal courts, federal courts and supreme court. |
| Number of People in Custody for Illicit Drugs-Related Criminal Charges | The first cut of data is estimated to be obtained in the spring of 2025, which will include data from 2015-2023. Data will be obtained annually from thereafter until 2027 |  | Canadian Correctional Services Survey (National [All Provinces]); Sourced via StatsCan: Collects data from correctional services programs in Canada, including characteristics of persons being supervised, legal hold status while in correctional services, offences and conditions related to court orders, events related to the person during supervision, and results of any needs assessments. Data includes average daily counts and intakes. |
|  |  |  |  |
