# Supplementary material for: The decriminalization of illicit drugs in British Columbia: a national evaluation protocol
Source: BMC Public Health. 2024 Oct 18;24:2879. doi: 10.1186/s12889-024-20336-9 (PMC11490149; doi:10.1186/s12889-024-20336-9)
Supplement: Supplementary file 4 — Supplementary Material 4: Appendix D. Sample interview guide for the qualitative sub-study with police. [file 12889_2024_20336_MOESM4_ESM.docx]

# Appendix D: Qualitative Police Interview Guide Sample

**Decriminalization of Illicit Substances in BC – Impacts on Policing Question Guide**

Thanks for taking the time to talk with me today. As a reminder, your responses today might be shared, but your identity will be kept confidential. You don’t have to answer any questions that you don’t want to or that may cause you discomfort. Information about support services can be provided if you’d like. The interview will take about 45 minutes to one hour of your time. At the end of the interview, I’ll ask you five short questions about your age, gender, that sort of thing.

***WE WOULD LIKE AUDIO RECORD THE INTERVIEW. IS IT OKAY IF WE BEGIN RECORDING NOW?***

***INDICATE WHETHER THE RESPONDENT/PARTICIPANTS HAS/HAVE PROVIDED VERBAL CONSENT FOR AUDIO RECORDING.*  Yes No**

The purpose of our conversation today is to understand your experience and views of BC’s Decriminalization Policy. Specifically, Health Canada granted BC a three-year exemption under Section 56(1) of the CDSA to permit adults to possess up to 2.5 grams of illicit drugs. This exemption came into force on January 31, 2023.

## Introduction

1. Can you start by telling me what your policing role is?
   1. If constable - So, thinking about your day-to-day work, what are some of the main ways that you come into contact with PWUD? What do these encounters look like?
   2. If specialized - What are some of the main ways you are engage in drug law enforcement? What does this engagement look like? Has this engagement changed at all since before decriminalization?

## General Views of the Exemption

1. What do you perceive the problem to be that the Decrim exemption is intended to address?
2. Do you think THIS exemption can solve or improve the problem you have identified?
   1. Is there another model or policy that would be better to address the problem you have identified?
3. What do you understand the policy objectives of this exemption to be?
   1. (if they don’t know, explain the objectives… If correct, just ask them how they feel about it) The policy objective for this exemption is to reduce harms associated with substance use including stigma, and to support PWUD in accessing health and social services and redirect them away from the criminal justice system. What do you think of this?
      1. What does stigma mean to you?

Implementation Questions of the Exemption

1. Overall, what do you think about BC’s exemption?
2. How did you first hear about? What did you think about it at first?
3. How were you prepared for the rollout of the exemption?
   - 1. Can you describe the training you received?
     2. Were you provided any resources, such as scales?
   1. Do you feel like police views were incorporated in the design/planning of the exemption?
      1. Ask generally (I.e. political design stage; implementation at the departmental level).
      2. Do you think there was a collaborative process in place for police to bring their perspective to the policy design?
4. Do you have any concerns about the exemption? If so, what are your concerns? Do you feel as though decriminalization has had an impact on policing? If so, has this been positive? Negative? If not, why do you think there has been no impact?
5. Do you feel as though decriminalization will have any impacts on policing in the future that may not have been realized/experienced yet?
6. As rollout has unfolded, have your views of decriminalization changed at all?
7. How are you measuring, if at all, the 2.5 gram limit?
   1. Are you strictly enforcing this 2.5 grams amount?
   2. Is there any discretion you use for amounts above 2.5 grams?
      1. NOTE this question might eliminate need to ask questions below RE change in discretion around possession.

## Changes in Interactions, charges, etc.

1. Since the Exemption was implemented in January 2023, how has the frequency or nature of your interactions with PWUD changed, if at all? If yes, in which ways? Can you provide an example of an interaction with PWUD since decriminalization, and how this is different than previous interactions? If no, can you describe why your interactions haven’t changed?
2. How, if at all, did your interactions with PWUD change in advance of decriminalization? How so? When? What prompted these changes?
3. How was your level of discretion with respect to simple possession changed following decriminalization, if at all? Can you describe an example of how your use of discretion has changed compared to how you would have applied it before decriminalization?
   1. Have there been any situations where you would still arrest and then recommend charges for simple possession? If so, can you please describe your reasoning?
4. Have there been any situations where you still seized drugs you deemed were for personal possession? If so, can you please describe your reasoning?
5. Have there been any situations in which you arrested but not charged a PWUD for simple possession? If so, can you please describe this type of encounter and your reasoning?
6. How has decriminalization changed how you respond to offences other than possession, if at all? If yes, how so?

How has the exemption impacted how you respond to or investigate drug trafficking?

1. Can you tell me about situations, if relevant, in which you enforced a bylaw or provincial offence against PWUD?
   1. For example, for public use, or another crime such as nuisance, loitering, etc.? If so, can you please describe this type of encounter and your reasoning?
   2. How do you think public drug consumption laws should be handled by police?
   3. How has your local community reacted to the changes?
   4. Have there been any local bylaws or policies related to drug use or PWUD that have been implemented since decriminalization? If so, can you describe these and their relation to/impact on decriminalization? What do you think of the bylaws?
      1. Have you heard of Bill 34, Restricting Public Consumption of Illegal Substances Act? ? If yes, how do you feel about it?
         1. If no, describe and ask “what do you think of it? (Description below)
            1. bans drug use in public and recreation-focused spaces including:

a six-metre radius from building entrances, including businesses and residential buildings;

within six metres of a bus stop;

within 15 metres of playgrounds, spray and wading pools, and skate parks; and

at parks, beaches and sports fields.

1. (if they talk about crown in the above, skip this question) How has the Exemption impacted your interactions with crown with respect to recommending charges for simple possession, if at all? If so, can you please describe these impacts?

## Operational Impacts

1. How has the Exemption impacted policing priorities for you? If it hasn’t had any impact, why do you think it hasn’t impacted priorities?
2. How has decriminalization impacted department policies and/or practices, if at all? If you think there has been no impact, why do you think it hasn’t impacted policies and/or practices?
3. How has decriminalization impacted your ability to focus on other policing matters, if at all?
   - 1. E.g. spend more time responding to serious crimes, etc.
4. How has it impacted your day-to-day work, if at all?

## Health System/Diversion

1. Are you aware of the information cards with information about harm and treatment services and resources which are supposed to be provided to PWUD if they ask for them or want them?
2. What are your thoughts on providing resource cards to PWUD? Do you think this is helpful or positive? How so?
3. What is your experience with providing resource cards?
4. How are the resource cards implemented and enforced within the department? Are there quotas or a specific number of cards you are supposed to provide? Do you have to keep track of how many you’ve provided to PWUD?

## Final Scoop

1. Is there anything else about decriminalization in BC that you want to say before we end the interview?
2. Do you know of anyone else who would be interested in participating? Can you pass on their contact info to us?

## Demographics

1. What city do you work in?
2. Can you tell me how old you are in years? _______ years. Prefer not to answer
3. What gender do you identify with?

Woman Man Trans man Trans woman Gender Non-Conforming Other: __________ Prefer not to answer

1. What ethnicity do you identify with?

White Black Hispanic/Latino Indian Middle Eastern South Asian Southeast Asian East Asian

First Nation, Metis, Inuit Indigenous Other Prefer not to answer

1. How long have you worked as a police officer?

________ years or months Prefer not to answer

1. How long have you worked as a police officer in this jurisdiction?

________ years/months Prefer not to answer

1. What is your rank?
2. Have you participated in a decriminalization study before?

Thank you for your time and sharing your thoughts today. We will likely be looking at policing perspectives again to understand how they may evolve over time (for e.g., 2, 3 years into the exemption). Would it be okay to follow-up with you following this interview if we have any further questions or to do another interview in 6-12 months’ time? Last question: Would you like to receive a copy of the research findings when the study is complete?
