# Supplementary material for: The decriminalization of illicit drugs in British Columbia: a national evaluation protocol
Source: BMC Public Health. 2024 Oct 18;24:2879. doi: 10.1186/s12889-024-20336-9 (PMC11490149; doi:10.1186/s12889-024-20336-9)
Supplement: Supplementary file 5 — Supplementary Material 5: Appendix E. Sample general population opinion polling survey. [file 12889_2024_20336_MOESM5_ESM.docx]

# Appendix E: General Population Opinion Polling (POP) Survey on Decriminalization of Illicit Drugs in British Columbia

Demographic Information (to be collected using Ipsos standard approaches)

Consent Blurb: *The next few questions are being asked on behalf of the Centre for Addiction and Mental Health (CAMH).  These questions examine the general public’s perceptions of the decriminalization of illicit drugs in British Columbia. Questions will ask about your awareness of the policy, your support or opposition to the policy, your feelings of community safety, and whether you think the policy will impact a number of different factors.*

*It is your choice whether you decide to answer these questions or not. While the research team doesn’t expect significant physical or mental risks associated with study participation, some participants may be reluctant to describe their perspectives. You do not have to answer any questions you do not feel comfortable with. The information learned may help inform public health policy making regarding the decriminalization of illicit substances.*

*If you have questions about this study, you can talk to the researcher who is in charge of the study at CAMH: C****ayley Russell*** *(Email:* [***OCRINTProject@camh.ca***](mailto:OCRINTProject@camh.ca)***).*** *If you have questions about your rights as a participant or about ethical aspects of this study, you can talk to the CAMH Chair of the Research Ethics Board (REB) at 416-535-8501 ext. 36798.*

Background

On January 31 2022, the province of British Columbia was granted a three-year exemption from the Controlled Drugs and Substances Act (CDSA) which allows adults aged 18+ in the province to legally possess a small amount of illegal drugs for personal possession.

Knowledge on Decriminalization

1. Based on your knowledge of the decriminalization of illegal drugs policy in BC, are the following statements true or false?

|  | True | False | Not sure | Prefer not to answer |
| --- | --- | --- | --- | --- |
| 1. Under the decriminalization policy in BC, individuals found in possession of a total of 2.5 grams of illicit drugs for personal use will not be criminally charged |  |  |  |  |
| 1. Police officers can still confiscate any amount of drugs found in someone’s possession |  |  |  |  |
| 1. Police officers are required to provide information and resources on community health and social services to people who use drugs upon request |  |  |  |  |
| 1. Possession of drugs for personal use is now legal, but using drugs in public is still illegal |  |  |  |  |
| 1. Decriminalization means that selling and/or distributing drugs is now legal in BC |  |  |  |  |

Impact of Decriminalization

Under decriminalization, adults are allowed to possess up to a cumulative total of 2.5 grams of opioids, cocaine/crack-cocaine, methamphetamine and MDMA for personal possession. Amounts carried above 2.5 grams will still be criminalized. The BC government’s stated goals of decriminalization are to reduce the harms associated with substance use, including stigma and criminalization, as well as to support people who use drugs to access health and social services, ultimately redirecting them away from the criminal justice system.

1. Please indicate your level of agreement or disagreement with the following statements regarding the potential impact of decriminalization of illegal drugs in BC.

|  | Strongly Disagree | Disagree | Neutral | Agree | Strongly Agree | Not Sure | Prefer not to answer |
| --- | --- | --- | --- | --- | --- | --- | --- |
| 1. Decriminalization will reduce the criminalization of people who use drugs in BC |  |  |  |  |  |  |  |
| 1. Decriminalization will reduce rates of drug overdoses |  |  |  |  |  |  |  |
| 1. Decriminalization will reduce the stigma associated with drug use |  |  |  |  |  |  |  |
| 1. Decriminalization has positively influenced my views of people who use drugs |  |  |  |  |  |  |  |
| 1. Decriminalization will encourage drug use experimentation |  |  |  |  |  |  |  |
| 1. Decriminalization has made me feel less safe in my community |  |  |  |  |  |  |  |
| 1. Decriminalization will decrease drug-related crimes in my community |  |  |  |  |  |  |  |
| 1. Decriminalization will reduce policing and law enforcement costs and resources |  |  |  |  |  |  |  |
| 1. Decriminalization will improve access to treatment and supports for people who use drugs |  |  |  |  |  |  |  |
| 1. Decriminalization is a positive step towards recognizing drug use as a health issue rather than a criminal issue |  |  |  |  |  |  |  |
| 1. I support the decriminalization of illicit drugs policy in BC |  |  |  |  |  |  |  |

1. Please describe what you feel the main benefit of the decriminalization policy is:
2. ___________________________
3. None
4. Not sure
5. Prefer not to answer
6. Please describe what your main concern with the decriminalization policy is
   1. ___________________________
   2. None
   3. Not sure
   4. Prefer not to answer
