# Supplementary material for: The decriminalization of illicit drugs in British Columbia: a national evaluation protocol
Source: BMC Public Health. 2024 Oct 18;24:2879. doi: 10.1186/s12889-024-20336-9 (PMC11490149; doi:10.1186/s12889-024-20336-9)
Supplement: Supplementary file 6 — Supplementary Material 6: Appendix F. Sample Harm Reduction and OAT service survey. [file 12889_2024_20336_MOESM6_ESM.docx]

# Appendix F: Mixed-Methods Harm Reduction and OAT Service Survey Samples

**BC OAT Service Utilization and Capacity Survey**

Thank you for taking the time to complete this survey. Your feedback will help us better understand the current state of OAT services in your area, and the potential impact of decriminalization on OAT services, which can help ensure that the needs of the community are being effectively met. On January 31 2022, the province of British Columbia was granted a three-year exemption from the Controlled Drugs and Substances Act (CDSA) which allows adults aged 18+ in the province to legally possess up to a cumulative total of 2.5 grams of opioids, cocaine/crack-cocaine, methamphetamine and MDMA for personal possession. Amounts carried above 2.5 grams will still be criminalized.

***This survey requires that the respondent completing it be able to speak to service operations (clientele, capacity, funding, etc.) and any potential changes since decriminalization. Please ensure the respondent has the required knowledge or can seek support from other staff to answer these questions.***

If your OAT site is integrated or affiliated with an existing/other organization/site, ***please respond only in regards to the OAT site component of your organization/service.***

****Section 1: General Information****

1. What is the name of the OAT site you are responding on behalf of:
2. Location (City/Region):
3. Regional Health Authority
   1. Northern Health Authority
   2. Interior Health Authority
   3. Vancouver Island Health Authority
   4. Vancouver Coastal Health Authority
   5. Fraser Health Authority
   6. Provincial Health Services Authority (PHSA)
   7. First Nations Health Authority (FNHA)
   8. Metis Nations Health Authority (MNHA)
   9. Other:_________
4. What is your position or role within the OAT site you are responding on behalf of?
   1. Program manager
   2. Intake/Administration
   3. Physician
   4. Nurse Practitioner
   5. Registered Nurse
   6. Director
   7. Other_____________
5. Is your OAT site integrated within or affiliated with an existing/other organization/site?
   1. Yes, it is integrated within an existing/other organization/site
   2. Yes, it is affiliated with an existing/other organization/site
   3. No, it is not integrated within an existing/other organization/site (i.e., it is a stand-alone service)
   4. No, it is not affiliated with an existing/other organization/site
   5. I don't know
6. When did your site open?
   1. Drop down menu with month/year

(For all respondents who select that their site opened after Jan 31, 2023, add skip logic to skip all questions asking about ‘changes since decriminalization’; also for these respondents, add the following two follow-up questions):

1. Did your site open because of the decriminalization policy?
   1. Yes
   2. No
   3. I don’t know
2. (Skip Logic) If yes, ***please describe how the decriminalization policy influenced the establishment of your OAT site***?
3. In an average week, please identify ***the days your OAT site is open, and for each selected, please specify the hours (e.g., 9-5) (check all that apply)***
   1. Monday ______
   2. Tuesday ______
   3. Wednesday _______
   4. Thursday ______
   5. Friday ______
   6. Saturday _______
   7. Sunday ______
4. (Skip logic) Since decriminalization (after January 31, 2023), ***have your OAT site days/hours changed***?
   1. Yes
   2. No
   3. I don’t know
5. (Skip logic) If yes, ***how have your OAT site operating days/hours changed*** (select all that apply)?
   1. Increased hours of operation
   2. Decreased hours of operation
   3. Increased days of operation
   4. Decreased days of operation
   5. Other: _________________________
   6. I don’t know

****Section 2: Clientele Profiles****

1. Is there ***a clientele focus for this OAT site?*** (e.g. women identifying only, pregnant people/parents, Indigenous-serving, LGBTQ2S+, youth-specific)
   1. Yes
   2. No
   3. I don’t know
2. (Skip logic) If yes, ***please describe the clientele focus***____________________________
3. For each of the following demographic categories, please *select the response that best reflects the* ***primary clientele who access OAT services at your OAT site***:
4. Age
   1. Under 18:
   2. 18-29:
   3. 30-49:
   4. 50+
   5. I don’t know
5. (Skip Logic) Since decriminalization, ***has there been a change in the average age of the primary clientele who access OAT services at your OAT site (e.g. increase in average age, decrease in average age)?***
   1. Yes
   2. No
   3. I don’t know
6. (Skip logic) If yes, ***please describe in which ways the average age of your primary clientele has changed***__________________________
7. Gender:
   1. Men
   2. Women
   3. Gender expansive (e.g., non-binary, gender queer, gender fluid)
   4. Approximate equal number of men/women/gender expansive
   5. I don’t know
8. Since decriminalization, ***has there been a change in the gender of the primary clientele (e.g., more men, more women)?***
   1. Yes
   2. No
   3. I don’t know
9. (Skip logic) If yes, ***please describe in which ways the gender of your primary clientele has changed***______________________

** For each of the following ethnicities, please identify ***the top three most common amongst clients who present to your OAT site*** (with 1 as the most common)*

1. Ethnicity:
   1. White (European descent):
   2. Black (African Caribbean Black/African Canadian Black)
   3. Indigenous (First Nations/Inuit/Metis)
   4. East/Southeast Asian (Chinese/Korean/Japanese/Filipino/Vietnamese/Thai)
   5. South Asian (East Indian/Pakistani/Bangladeshi/Sri Lankan/Indo-Caribbean)
   6. Middle Eastern (Arab/Persian/Afghan/Egyptian/Iranian/Lebanese/Turkish)
   7. Latino (Latin American/Hispanic descent)
   8. Other (please specify)
   9. I don’t know
2. (Skip logic) Since decriminalization, ***has there been a change in the ethnicity of your primary clientele (e.g., more people who identify as Indigenous or people of color, less people who identify as Indigenous or people of color)?***
   1. Yes
   2. No
   3. I don’t know
3. (Skip logic) If yes, ***please describe in which ways the ethnicity of your primary clientele has changed***______________________

****Section 3: OAT Information****

1. Please specify ***the type(s) of OAT prescribed at your OAT site*** (select all that apply):
   1. Methadone (Methadose/Metadol-D)
   2. Buprenorphine/naloxone (Suboxone)
   3. Slow Release Oral Morphine (SROM; Kadian)
   4. Injectable OAT (iOAT; diacetylmorphine)
   5. Tablet Injectable OAT (TiOAT); hydromorphone/Dilaudid)
   6. Transdermal/patch fentanyl
   7. Extended-release buprenorphine (Sublocade, Brixadi)
   8. Other:____________________
2. (Skip Logic from services selected) Based on the types of OAT that you selected that your organization currently offers, ***please identify the top 3 most frequently prescribed OAT formulations*** (i.e., 1-3, with 1 being the most prescribed)
   1. 1_____________
   2. 2_____________
   3. 3_____________
3. Does your OAT site prescribe ***take-home/unwitnessed doses (carries)***?
   1. Yes
   2. No
   3. I don’t know
4. (Skip logic) If yes, since decriminalization***, have you observed any changes in the frequency of prescriptions for take-home/unwitnessed doses of OAT at your site?***
   1. Yes, increased significantly
   2. Yes, increased slightly
   3. No, remained the same
   4. Yes, decreased slightly
   5. Yes, decreased significantly
   6. I don’t know
5. Does your OAT site have ***specific eligibility criteria for clients to receive OAT?***
   1. Yes
   2. No
   3. I don’t know
6. (Skip Logic), If yes, ***what is the eligibility criteria (select all that apply)?***
   1. Opioid use disorder diagnosis (i.e., DSM-5-TR)
   2. Other standardized tests (e.g., clinical opioid withdrawal scale [COWS], drug abuse screening test [DAST] )
   3. History of substance use addiction
   4. Urinalysis testing
   5. Age
   6. Pregnancy status
   7. Other (please specify)____________________
   8. I don’t know

****Section 5: Service Capacity and Waitlists****

1. On average, ***how many clients are on OAT at your site each month (including clients that come in daily as well as clients with extended prescriptions)?***
   1. Less than 50
   2. 50-100
   3. 101-200
   4. 201-300
   5. Over 400
   6. I don’t know
2. (Skip logic) Since decriminalization, ***has the average number of clients accessing your OAT site in a given month changed (e.g., increased, decreased)?***
   1. Yes
   2. No
   3. I don’t know
3. (Skip logic) If yes, ***how has the number of clients on OAT in a given month changed***?
   1. Increased significantly
   2. Increased slightly
   3. Decreased slightly
   4. Decreased significantly
   5. I don’t know
4. Is there ***currently a waitlist to receive OAT at your site?***
   1. Yes
   2. No
   3. I don’t know
5. (Skip logic) If yes, please provide the approximate current waitlist time
   1. Less than a week
   2. 1-2 weeks
   3. 2-4 weeks
   4. 1 month
   5. 1-2 months
   6. 2+ months
   7. Other_____________________
6. (Skip logic) If yes, ***have there been any changes to the waitlist to receive OAT since decriminalization?***
   1. Yes
   2. No
   3. I don’t know
7. (Skip logic) If yes, ***how has the waitlist to receive OAT changed?***
   1. Increased significantly
   2. Increased slightly
   3. Decreased slightly
   4. Decreased significantly
   5. I don’t know
8. Does your OAT site ***offer drop-in services to receive OAT?***
9. Yes
10. No
11. I don’t know
12. (skip logic) If yes, ***what is the average wait time that clients have to wait to receive OAT when they drop in?***
    1. Less than an hour
    2. 1-2 hours
    3. 2-3 hours
    4. More than 3 hours
    5. I don’t know
    6. Other _____________

*****Section 7: Treatment Retention******

1. On average***, how long do your clients typically stay on OAT?***
2. Less than 1 month
3. 1 to 3 months
4. 3 to 6 months
5. 6 months to 1 year
6. More than 1 year
7. Other
8. I don’t know
9. (Skip logic) Since decriminalization, ***has the average length of time clients typically stay on OAT changed?***
   1. Yes
   2. No
   3. I don’t know
10. (skip logic) If yes, ***how has the average length of time clients typically stay on OAT changed?***
    1. Increased significantly
    2. Increased slightly
    3. Decreased slightly
    4. Decreased significantly
    5. I don’t know

****Section 8: Resources****

1. Please select your ***OAT site’s primary funding source***:
   1. Non-government organization
   2. Municipal
   3. Provincial (funding from health authorities)
   4. Federal
   5. Fundraising/donations
   6. Other (please specify):__________________________________
   7. I don’t know
2. (Skip logic) Since decriminalization, ***has your OAT site’s funding source changed***?
   1. Yes
   2. No
   3. I don’t know
3. (Skip logic) If yes, ***please describe how your OAT site’s funding source has changed***: _______________________________________________________________________
4. Does your site charge a fee for patients to receive OAT?
   1. Yes
   2. No
   3. I don’t know
5. (Skip logic) If yes, ***how much of the full cost of OAT do patients pay?***
   1. The full cost
   2. Partial cost
   3. I don’t know
6. Please select the type and provide the number of staff members that are ***currently employed within your OAT site*** (full- and part-time, contract-based; if employees hold more than one role, please indicate their primary role):
   1. Physicians, Number:_____
   2. Registered Nurses, Number:____
   3. Nurse practitioners, Number:_____
   4. Pharmacists, Number:_____
   5. Administrative personnel, Number:_____
   6. Addiction counselors/therapists, Number:_____
   7. Other (Please specify), Number:_____
7. Please identify ***the types of providers prescribing OAT at your OAT site*** (check all that apply):
   1. Physicians
   2. Registered nurses
   3. Nurse Practitioners (NP)
   4. Pharmacists
   5. Other_________________
8. (Skip logic) Since decriminalization, has the ***demand on staff changed at your OAT site***? (increased or decreased workload, required training, emotional support)?
   1. Yes
   2. No
   3. I don’t know
9. (Skip logic), If demand on staff has changed, ***please specify in what areas the demand on your OAT site’s staff has changed*** (select all that apply):
   1. Workload
   2. Client intake
   3. Administrative tasks
   4. Training and development
   5. Client interactions
   6. Scheduling
   7. Other (please specify): _________________
   8. I don’t know

1. (Skip logic) For each area selected, ***please describe how the demand on your OAT site’s staff has changed***:
   1. Increased significantly
   2. Increased slightly
   3. Decreased slightly
   4. Decreased significantly
   5. I don’t know
2. Does your current ***staffing meet your OAT site’s demand***?
   1. Yes
   2. No
   3. I don’t know
3. Do your ***current resources (e.g. supplies, space, equipment) meet your OAT site’s demand?***
   1. Yes
   2. No
   3. I don’t know
4. (Skip logic) Since decriminalization, has the ***demand on your OAT site’s resources changed?*** (e.g., supplies, space, equipment)?
   1. Yes
   2. No
   3. I don’t know
5. (Skip logic), If yes, ***please specify in what areas the demand on your OAT site’ resources has changed*** (select all that apply):
   1. Medication
   2. Medical/harm reduction supplies (e.g., injection supplies, naloxone)
   3. Space/facility usage
   4. Educational materials
   5. Technological equipment
   6. Other (please specify): _________________
6. (Skip logic) For each area selected, ***please describe how the demand on your OAT site’s resources has changed***:
   1. Increased significantly
   2. Increased slightly
   3. Decreased slightly
   4. Decreased significantly
   5. I don’t know
7. Did your ***OAT site/staff receive formal decriminalization training***?
   1. Yes
   2. No
   3. I don’t know
8. (Skip logic) If no, do you feel as though ***your OAT site/staff would benefit from decriminalization training***?
   1. Yes
   2. No
   3. I don’t know
9. (Skip logic) If yes, ***what type of decriminalization training did your OAT site/staff receive?*** (select all that apply)
   1. Online/Modules
   2. In-person
   3. Other_____
   4. I don’t know
10. (Skip logic) If yes, what was the ***length of decriminalization training (e.g., one hour, one day)____________________________________________***
11. (Skip logic) If yes, ***how was the decriminalization training conducted (e.g., who provided the training, any additional details)?_________________________________***
12. (Skip logic) If yes, ***do you feel as though the kind of decriminalization training provided adequate knowledge on the decriminalization policy to your OAT site/staff?***
    1. Yes
    2. No
    3. I don’t know

****Section 9: Referral Pathways****

1. Does your OAT site ***have formal (i.e., required mandate or formal collaboration) referral pathways with other sites/services***?
   1. Yes
   2. No
   3. I don’t know
2. (Skip logic) If yes, ***please identify the nature of the referrals*** (select all that apply):
   1. Other services refer clients to us
   2. We refer clients to other services
   3. I don’t know
3. (Skip logic) If your site refers clients to other services, ***which treatment services or programs does your OAT site commonly refer clients to for additional support or treatment? (select all that apply)***
   1. Inpatient detoxification/withdrawal management programs
   2. Outpatient detoxification/withdrawal management programs
   3. Residential treatment programs
   4. Safe consumption services/overdose prevention services
   5. Drug checking services
   6. Safe supply prescribers
   7. Naloxone distribution services
   8. Sexually transmitted and blood-borne infection (STBBI) testing
   9. General practitioners/physicians/doctors
   10. Substance use counseling
   11. Mental health counseling
   12. Indigenous-specific services (e.g., Elders and/or traditional healers, smudging)
   13. Peer support services
   14. Social and family support services (shelter/housing, food, drop-in, washing/showering facilities)
   15. Other (please specify): _________________
4. (Skip logic) If your organization refers clients to other services, since decriminalization, ***how has the frequency of your OAT site’s referrals to other services changed?***
   1. Increased significantly
   2. Increased slightly
   3. Remained the same
   4. Decreased slightly
   5. Decreased significantly
   6. I don’t know
5. Do your clients commonly mention ***(formally such as during intake, or informally, during conversation)*** the decriminalization policy as a reason for accessing your OAT site?
   1. Yes
   2. No
   3. I don’t know
6. (Skip logic), If yes, how often do clients mention ***(formally such as during intake, or informally, during conversation)*** the decriminalization policy as a reason for accessing your OAT site?
   1. Often
   2. Occasionally
   3. Rarely
   4. Never
   5. I don’t know
7. Do your clients ***reference the decriminalization police resource cards as a reason or influencing factor for accessing your OAT site?***
8. Yes
9. No
10. I don’t know
11. (Skip logic), If yes, how often do clients ***reference the police resource cards as a reason or influencing factor for accessing your OAT site?***
12. Often
13. Occasionally
14. Rarely
15. Never
16. I don’t know

****Section 10: Community Impacts****

1. Does your ***OAT site receive drug alerts?***
   1. Yes, staff are subscribed to receive text-based drug alerts
   2. Yes, we receive drug alerts by email
   3. No
   4. I don’t know
2. (skip logic), If yes, ***does your OAT site share drug alerts and/or information contained in drug alerts with clients?***
   1. Yes
   2. No
   3. I don’t know
3. Does your OAT site ***experience any law enforcement/police activity on or near the premises?***
   1. Yes
   2. No
   3. I don’t know
4. (Skip logic) Since decriminalization, ***have there been any changes in law enforcement/police activity on or near your OAT site’s premises?***
   1. Increased law enforcement/police activity
   2. No changes
   3. Decreased law enforcement/police activity
   4. I don’t know
5. (Skip logic for sites that opened post-decriminalization) What has ***the community response to the opening of your OAT site been***?
   1. Highly positive
   2. Somewhat positive
   3. Neutral
   4. Somewhat negative
   5. Highly negative
   6. I don’t know

****Section 11: Guidelines ****

1. Which of the following ***OAT prescribing guidelines does your OAT site adhere to? (select all that apply)***
   1. British Columbia Centres for Substance Use (BCCSU) Opioid Use Disorder Clinical Guidelines
   2. Centre for Addiction and Mental Health (CAMH) Synthesis of Canadian Guidelines
   3. Canadian Research Initiative in Substance Misuse (CRISM) National Opioid Use Disorder Guideline
   4. Regulatory Body (i.e., College of Physicians and Surgeons of BC, BC College of Nurses and Midwives, etc.)
   5. Mentoring, Education, Clinical Tools for Addiction: Partners in Health Integration (META:PHI) Approaches for Methadone Carries
   6. None
   7. Other:___________________
   8. I don’t know

******Section 12: Final Thoughts******

1. Are there any other ***notable changes or challenges your OAT site has faced since decriminalization that haven't been addressed in the previous questions?***
   1. Yes
   2. No
   3. I don’t know
2. (Skip logic) If yes, ***please describe the changes or challenges your OAT site has faced since decriminalization***__________________________

Thank you for completing the survey. Your insights are valuable in ensuring that OAT services are accessible and effective for all who need them, and to truly understand the impact the decriminalization policy has had on OAT services in BC.

(These questions will appear on a separate form/page to separate PHI from responses)

1. Would you like to receive the $25 Amazon E-gift card for participating?
   1. Yes
   2. No
2. (Skip logic) If yes, ***please provide your email address*** ____________________________________________________________
3. Are you ***interested in being contacted to do a one-on-one key-informant interview over the phone where we will ask you additional questions regarding your organization and any changes since decriminalization?***
   1. Yes
   2. No

If no (b) to Q79 and yes (a) to Q81:

1. (Skip logic) If yes, ***please provide your email address/phone number*** ____________________________________________________________

**BC Harm Reduction Service Utilization and Capacity Survey**

Thank you for taking the time to complete this survey. Your feedback will help us better understand the current state of harm reduction services in your area, and the potential impact of decriminalization on harm reduction services, which can help ensure that the needs of the community are being effectively met.

On January 31 2022, the province of British Columbia was granted a three-year exemption from the Controlled Drugs and Substances Act (CDSA) which allows adults aged 18+ in the province to legally possess up to a cumulative total of 2.5 grams of opioids, cocaine/crack-cocaine, methamphetamine and MDMA for personal possession. Amounts carried above 2.5 grams will still be criminalized.

***This survey requires that the respondent completing it be able to speak to service operations (e.g., clientele, capacity, funding) and any potential changes since decriminalization. Please ensure the respondent has the required knowledge or can seek support from other staff to answer these questions.***

If your harm reduction site is integrated or affiliated with an existing/other organization/site, ***please respond only in regards to the harm reduction site component of your organization/service***

****Section 1: General Information****

1. What is the ***name of the harm reduction site you are responding on behalf of:***
2. What is your position or role within the harm reduction site you are responding on behalf of?
   1. Program manager
   2. Intake worker
   3. Director
   4. Harm reduction support worker
   5. Nursing staff
   6. Other __________
3. What is the primary purpose of your harm reduction site? (Please check the answer that best describes the main purpose of your organization, even if your organization offers multiple services):
   1. Harm reduction supplies distribution (e.g., safer use kits)
   2. Safe consumption/injection/inhalation services
   3. Overdose prevention services
   4. Drug checking
   5. Safe supply prescriptions
   6. Community syringe/paraphernalia pick-up
   7. Naloxone distribution
   8. Opioid agonist treatment (OAT) prescriptions
   9. Sexually transmitted and blood-borne infection (STBBI) testing
   10. Mobile outreach
   11. Clinical care/healing (e.g. wound care, vaccinations, primary care)
   12. Substance use counseling
   13. Health education
   14. Detox/withdrawal management
   15. Indigenous-specific services (e.g., Elders and/or traditional healers, smudging)
   16. Social support (shelter/housing, food, drop-in, washing/showering facilities)
   17. Other (please specify): _________________
4. Is your harm reduction site integrated within or affiliated with an existing/other organization/site?
   1. Yes, it is integrated within an existing/other organization/HR site
   2. Yes, it is affiliated with an existing/other organization/HR site
   3. No, it is not integrated within an existing/other organization/HR site (i.e., it is a stand-alone service)
   4. No, it is not affiliated with an existing/other organization/HR site
   5. I don't know
5. Harm reduction site location (City/Region):
6. Regional Health Authority
   1. Northern Health Authority
   2. Interior Health Authority
   3. Vancouver Island Health Authority
   4. Vancouver Coastal Health Authority
   5. Fraser Health Authority
   6. Provincial Health Services Authority (PHSA)
   7. First Nations Health Authority (FNHA)
   8. Metis Nations Health Authority (MNHA)
   9. Other:_____________________
7. Is your ***harm reduction site registered with the BC Centres for Disease Control (BCCDC) (i.e., orders harm reduction supplies directly from the BCCDC)***?
   1. Yes
   2. No
8. When did your harm reduction site open?
   1. Drop down menu with month/year

(For all respondents who select that their site opened after Jan 31 2023, add skip logic to skip all questions asking about ‘changes since decriminalization’; also, for these respondents, add the following two follow-up questions):

1. Did your harm reduction site ***open because of the decriminalization policy?***
   1. Yes
   2. No
   3. I don’t know
2. (Skip Logic) If yes, ***please describe how the decriminalization policy influenced the establishment of your OAT site***?
3. In an average week, please ***identify the days your harm reduction site is open, and for each selected, please specify the hours*** (e.g., 9-5) (check all that apply)
   1. Monday ______
   2. Tuesday ______
   3. Wednesday _______
   4. Thursday ______
   5. Friday ______
   6. Saturday _______
   7. Sunday ______
4. (Skip logic) Since decriminalization (after January 31, 2023), ***have your operating days/hours changed***?
   1. Yes
   2. No
   3. I don’t know
5. (Skip logic) If yes, ***how have your operating days/hours changed*** (select all that apply)?
   1. Increased hours of operation
   2. Decreased hours of operation
   3. Increased days of operation
   4. Decreased days of operation
   5. Other: _________________________
   6. I don’t know

****Section 2: Clientele Profiles****

1. Is there ***a clientele focus for this site?*** (e.g. women identifying only, pregnant people/parents, Indigenous-serving, LGBTQ2S+, youth-specific)
   1. Yes
   2. No
   3. I don’t know
2. (Skip logic) If yes, ***please describe the clientele focus***____________________________
3. For each of the following demographic categories, please select the response that best reflects the ***primary clientele who present to your harm reduction site***:
4. Age
   1. Under 18:
   2. 18-29:
   3. 30-49:
   4. 50+
   5. I don’t know
5. (Skip logic) Since decriminalization, ***has there been a change in the average age of your primary clientele (e.g. increase in average age, decrease in average age)?***
   1. Yes
   2. No
   3. I don’t know
6. (Skip logic) If yes, ***please describe in which ways the average age of your primary clientele has changed***__________________________
7. Gender:
   1. Men
   2. Women
   3. Gender expansive (e.g., non-binary, gender queer, gender fluid)
   4. Approximate equal number of men/women/gender expansive
   5. I don’t know
8. (Skip logic) Since decriminalization, ***has there been a change in the gender of your clientele (e.g., more men, more women)?***
   1. Yes
   2. No
   3. I don’t know
9. (Skip logic) If yes, ***please describe in which ways the gender of your clientele has changed (e.g., more men, more women)?***__________________________

** For each of the following ethnicities, please *identify* ***the top three most common amongst clients who present to your harm reduction site*** (with 1 as the most common)*

1. Ethnicity:
   1. White (European descent):
   2. Black (African Caribbean Black/African Canadian Black)
   3. Indigenous (First Nations/Inuit/Metis)
   4. East/Southeast Asian (Chinese/Korean/Japanese/Filipino/Vietnamese/Thai)
   5. South Asian (East Indian/Pakistani/Bangladeshi/Sri Lankan/Indo-Caribbean)
   6. Middle Eastern (Arab/Persian/Afghan/Egyptian/Iranian/Lebanese/Turkish)
   7. Latino (Latin American/Hispanic descent)
   8. Other (please specify)
   9. I don’t know
2. (Skip logic) Since decriminalization, ***has there been a change in the ethnicity of your primary clientele (e.g., more people who identify as Indigenous or people of color, less people who identify as Indigenous or people of color)?***
   1. Yes
   2. No
   3. I don’t know
3. (Skip logic) If yes, ***please describe in which ways the ethnicity of your primary clientele has changed***__________________________

** For each of the following substance use categories, please ***rank the top three*** most commonly used substances by your clients (with 1 as the most common), as well as the ***primary route of administration*** for each)**

1. Illicit substances ***primarily used by your harm reduction site’s clientele***:
   1. Illegal/street-sourced opioids (fentanyl, down, heroin)
   2. Non-prescribed opioids (hydromorphone/Dilaudid, morphine/Kadian, etc.)
   3. Methamphetamine
   4. Powder cocaine
   5. Crack-cocaine
   6. Stimulant and opioid combinations (Speedball, Goofball, etc.)
   7. Ecstasy/MDMA
   8. Non-prescribed benzodiazepines (Valium, Xanax, Ativan)
   9. Hallucinogens/Disassociatives (Shrooms, Psilocybin, DMT, PCP, Ketamine)
   10. Other (please specify): _________________
   11. I don’t know
2. (Skip Logic) For each ranked response, ***please specify the primary route of administration***
   1. Inhalation
   2. Injection
   3. Oral
   4. Nasal
   5. Other (please specify)
   6. I don’t know
3. (Skip logic) Since decriminalization, ***have you observed a change in the primary substances used by your clientele***?
   1. Yes
   2. No
   3. I don’t know
4. (Skip logic) If yes, ***please describe in which ways the primary substances used by your clientele have changed (e.g., using more methamphetamine and less down)***__________________________
5. (Skip logic) Since decriminalization, ***have you observed a change in the frequency of substance use by your regular clientele (i.e., your clients are using more or less frequently than before)***?
   1. Yes
   2. No
   3. I’m seeing different clients than before or we don’t have regular clients
   4. I don’t know
6. (Skip logic) If yes, ***please describe how your regular clientele’s frequency of substance use has changed***
   1. Clients are using substances more frequently
   2. Clients are using substances less frequently
   3. I don’t know

****Section 3: Service Utilization****

1. Aside from the main purpose of your harm reduction site that you already identified at the beginning of this survey, ***what other services does your harm reduction site currently offer***? (check all that apply):
   1. Harm reduction supplies distribution (e.g., safer use kits, etc.)
   2. Safe consumption injection services
   3. Safe consumption injection and inhalation services
   4. Overdose prevention services
   5. Drug checking
   6. Safe supply prescriptions
   7. Community syringe/paraphernalia pick-up
   8. Naloxone distribution (including take-home naloxone distribution/training)
   9. Opioid agonist treatment (OAT) prescriptions (including iOAT and TiOAT)
   10. Sexually transmitted and blood-borne infection (STBBI) testing
   11. Mobile outreach
   12. Clinical care/healing (e.g. wound care, vaccinations, primary care)
   13. Substance use counseling
   14. Mental health counseling
   15. Health education
   16. Detox/withdrawal management
   17. Indigenous-specific services (e.g., Elders and/or traditional healers, smudging)
   18. Peer support
   19. Social and family support (shelter/housing, food, drop-in, washing/showering facilities)
   20. Other (please specify): _________________
   21. I don’t know
2. Are any of the services offered specifically ***in response to the decriminalization policy?***
   1. Yes
   2. No
   3. I don’t know
3. (Skip logic) If yes, please ***list the services that were directly in response to the decriminalization policy***_________________________________________
4. Are any of the services offered ‘***pop-up’ services (i.e., a temporary/emergency solution that is not formally sanctioned/approved)?***
   1. Yes
   2. No
   3. I don’t know
5. If yes, please ***list the ‘pop-up’ services offered***_________________________________
6. Is your harm reduction site planning to ***expand or modify your services in the near future based on the decriminalization policy?***
   1. Yes
   2. No
   3. I don’t know
7. (Skip logic) If yes, please specify ***the types of changes your harm reduction site is considering***___________________________________________________________

1. On average, ***how many clients access your harm reduction site in a given month***?
   1. Less than 50
   2. 50-100
   3. 101-200
   4. 201-300
   5. Over 400
   6. I don’t know
2. (Skip logic) Since decriminalization, ***has the average number of clients accessing your harm reduction site in a given month changed (e.g., increased, decreased)?***
   1. Yes
   2. No
   3. I don’t know
3. (Skip logic) If yes, ***how has the number of clients accessing your harm reduction site in a given month changed***?
   1. Increased significantly
   2. Increased slightly
   3. Decreased slightly
   4. Decreased significantly
   5. I don’t know
4. Have you ever ***had to turn clients away or ask clients to wait in a waiting room due to a lack of capacity or resources to accommodate them***?
   1. Yes
   2. No
   3. I don’t know
5. (Skip logic) If yes, ***how often does this happen:***
6. Often
7. Occasionally
8. Rarely
9. Never
10. I don’t know

****Section 4: Resources****

1. Please select your ***harm reduction site’s primary funding source***:
   1. Non-government organization
   2. Municipal
   3. Provincial (funding from health authorities)
   4. Federal
   5. Fundraising/donations
   6. Other (please specify):_________________________________
   7. I don’t know
2. (Skip logic) Since decriminalization, ***has your harm reduction site’s funding source changed***?
   1. Yes
   2. No
   3. I don’t know
3. (Skip logic) If yes, ***please describe how your harm reduction site’s funding source has changed***: ________________________________________________________________________
4. What is your harm reduction site’s ***annual operating budget***? (You can provide an estimate if exact figures are not available)
   1. Less than $100,000
   2. $100,000 - $500,000
   3. $500,000 - $1 million
   4. Over $1 million
   5. I don’t know
5. (Skip logic) Since decriminalization, ***has your harm reduction site’s annual operating budget changed (e.g. increased, decreased)?***
   1. Yes
   2. No
   3. I don’t know
6. (Skip logic) If yes, ***how has your harm reduction site’s annual operating budget changed***?
   1. Increased significantly
   2. Increased slightly
   3. Decreased slightly
   4. Decreased significantly
   5. I don’t know
7. Please select the type and provide the number of staff members that are ***currently employed within your harm reduction site*** (full- and part-time, and contract-based; if employees hold more than one role, please indicate their primary role):
   1. Intake workers, Number:_____
   2. Registered Nurses, Number:_____
   3. Nurse practitioners, Number:_____
   4. Medical doctors, Number:_____
   5. Outreach workers, Number:_____
   6. Peer supporters, Number:_____
   7. Social workers, Number:_____
   8. Administrative personnel, Number:_____
   9. Managers, Number:_____
   10. Frontline/Overdose Response Workers, Number:______
   11. Other (Please specify), Number:_____
8. (Skip logic) Since decriminalization, has the ***demand on your harm reduction site’s staff changed***? (e.g., increased or decreased workload, required training, emotional support)?
   1. Yes
   2. No
   3. I don’t know
9. (Skip logic), If demand on staff has changed, ***please specify in what areas the demand on your harm reduction site’s staff has changed*** (select all that apply):
   1. Workload
   2. Client intake
   3. Administrative tasks
   4. Training and development
   5. Community outreach
   6. Overdose prevention/response
   7. Client interactions
   8. Scheduling
   9. Other (please specify): _________________
   10. I don’t know
10. (Skip logic) For each area selected, ***please describe how the demand on your harm reduction site’s staff has changed***?
    1. Increased significantly
    2. Increased slightly
    3. Decreased slightly
    4. Decreased significantly
    5. I don’t know
11. Does your current ***staffing meet your harm reduction site’s demand***?
    1. Yes
    2. No
    3. I don’t know
12. Do your ***current resources (e.g. supplies, space, equipment) meet your OAT site’s demand?***
    1. Yes
    2. No
    3. I don’t know
13. (Skip logic) Since decriminalization, has the ***demand on your harm reduction site’s resources changed?*** (e.g., supplies, space, equipment)?
    1. Yes
    2. No
    3. I don’t know
14. (Skip logic), If yes, ***please specify in what areas the demand on your harm reduction site’s resources has changed*** (select all that apply):
    1. Medical/harm reduction supplies (e.g., injection supplies, naloxone)
    2. Space/facility usage
    3. Educational materials
    4. Technological equipment
    5. Transportation (e.g., mobile units)
    6. Other (please specify): _________________
15. (Skip logic) For each area selected, ***please describe how the demand on your harm reduction site’s resources has changed:***
    1. Increased significantly
    2. Increased slightly
    3. Decreased slightly
    4. Decreased significantly
    5. I don’t know
16. Did your ***harm reduction site/staff receive formal decriminalization training***?
    1. Yes
    2. No
    3. I don’t know
17. (Skip logic) If no, do you feel as though ***your harm reduction site/staff would benefit from decriminalization training***?
    1. Yes
    2. No
    3. I don’t know

1. (Skip logic) If yes, ***what type of decriminalization training did your harm reduction site/staff receive? (select all that apply)***
   1. Online/Modules
   2. In-person
   3. Other_____
   4. I don’t know
2. (Skip logic) If yes, what was the ***length of the decriminalization training (e.g., one hour, one day)____________________________________________***
3. (Skip logic) If yes, ***how was the decriminalization training conducted (e.g., who provided the training, any additional details)?_________________________________***
4. (Skip logic) If yes, ***do you feel as though the kind of decriminalization training provided adequate knowledge on the decriminalization policy to your harm reduction site/staff?***
5. Yes
6. No
7. I don’t know

****Section 5: Referral Pathways****

1. Do your clients commonly ***mention (formally such as during intake, or informally, during conversation) the decriminalization policy as a reason for accessing your harm reduction site?***
   1. Yes
   2. No
   3. I don’t know

1. (Skip logic), If yes, ***How often do clients mention (formally such as during intake, or informally, during conversation) the decriminalization policy as a reason for accessing your harm reduction site***?
   1. Often
   2. Occasionally
   3. Rarely
   4. Never
   5. I don’t know
2. Do your clients ***reference the decriminalization police resource cards as a reason or influencing factor for accessing your harm reduction site?***
   1. Yes
   2. No
   3. I don’t know
3. (Skip logic), If yes, how often do clients ***reference the police resource cards as a reason or influencing factor for accessing your harm reduction site?***
4. Often
5. Occasionally
6. Rarely
7. Never
8. I don’t know

****Section 6: Community Impacts****

1. Does your harm reduction site ***receive drug alerts?***
   1. Yes, staff are subscribed to receive text-based drug alerts
   2. Yes, we receive drug alerts by email
   3. No
   4. I don’t know
2. (skip logic), If yes, does your harm reduction site ***share drug alerts and/or information contained in drug alerts with clients?***
   1. Yes
   2. No
   3. I don’t know
3. Does your harm reduction site ***experience any law enforcement/police activity on or near your premises?***
   1. Yes
   2. No
   3. I don’t know
4. (skip logic) If yes, since decriminalization, ***have there been any changes in law enforcement/police activity on or near your harm reduction site’s premises?***
   1. Increased law enforcement/police activity
   2. No changes
   3. Decreased law enforcement/police activity
   4. I don’t know
5. (Skip logic for sites that opened post-decriminalization) What has ***the community response to the opening of your harm reduction site been***?
   1. Highly positive
   2. Somewhat positive
   3. Neutral
   4. Somewhat negative
   5. Highly negative
   6. I don’t know

****Section 7: Final Thoughts****

1. Are there any other ***notable changes or challenges your harm reduction site has faced since decriminalization that haven't been addressed in the previous questions?***
2. Yes

b. No

c. I don’t know

1. (Skip logic) If yes, ***please describe the changes or challenges your harm reduction site has faced since decriminalization***__________________________

Thank you for completing the survey. Your insights are valuable in ensuring that harm reduction services are accessible and effective for all who need them, and to truly understand the impact the decriminalization policy has had on harm reduction services in BC. If you would like to provide your email address to receive the E-gift card or to put your name forward to be contacted for a follow-up interview, please click the link below.

(These questions will appear on a separate form/page to separate PHI from responses)

1. Would you like to receive the $25 Amazon E-gift card?
   1. Yes
   2. No
2. (Skip logic) If yes, ***please provide your email address*** ____________________________________________________________
3. Are you ***interested in being contacted to do a one-on-one key-informant interview over the phone where we will ask you additional questions regarding your organization and any changes since decriminalization?***
   1. Yes
   2. No

If no (b) to Q76 and yes (a) to Q78:

1. (Skip logic) If yes, ***please provide your email address/phone number*** ____________________________________________________________
